# Cardiovascular event rate modifies response to pharmacologic LDL-C lowering in primary prevention: implications of a systematic review and meta-analysis for clinical practice

**DOI:** 10.1016/j.ajpc.2026.101655

**Published:** 2026-05-25

**Authors:** Irene Karungi, Christophe A.T. Stevens, Julia Brandts, Kausik K Ray

**Affiliations:** a Department of Primary Care and Public Health, School of Public Health, Imperial College London, London, United Kingdom; b Department of Medicine I, RWTH University Hospital Aachen, Aachen, Germany

**Keywords:** Primary prevention, Lipid-lowering therapy, Major adverse cardiovascular event (mace), Low-density lipoprotein cholesterol (ldl-c)

## Abstract

**Background:**

LDL-C lowering is often delayed in lower-risk primary-prevention settings as absolute benefits appear modest. Trial evidence for greater relative benefits from pharmacologic LDL-C lowering in lower-risk individuals, supporting genetic studies, could strengthen the rationale for initiating LDL-C-lowering therapies at lower-risk levels.

**Objectives:**

To quantify i) how RRR for 3P-MACE per 1mmol/L LDL-C-lowering varies by baseline risk, ii) the absolute LDL-C reduction required to achieve 25 % RRR at varying risk thresholds.

**Methods:**

Systematic review and meta-analysis using EMBASE, MEDLINE, and CENTRAL searches for randomized, placebo-controlled lipid-lowering trials in populations with no or low (<20 %) prior atherosclerotic cardiovascular disease prevalence, reporting 3P-MACE (cardiovascular death, non-fatal myocardial infarction, non-fatal stroke). Effect modification of placebo event rate on RRR/1mmol/L was assessed using mixed-effects meta-regression. A second meta-regression plotted the absolute LDL-C reduction associated with 25 % RRR across event-rates.

**Results:**

17 trials (105,879 participants) reporting 6076 3P-MACE were included (12 statins only, 5 non-statins); mean age 63.0y, median follow-up 4.4y. LDL-C reduction ranged from 0.38–1.95 mmol/L and placebo event-rate ranged from 0.52 %/year-3.78 %/year. RRR per 1mmol/L LDL-C reduction attenuated from 36 % at 1 %/year event-rate to 13 % at 3 %/year (*p* < 0.0001). Absolute LDL-C reductions required to achieve 25 % RRR increased with baseline-risk, ranging from 0.36 mmol/L at 1 %/year-risk to 3.09 mmol/L at 3 %/year-risk (*p* = 0.0001).

**Conclusion:**

Lower-risk primary prevention populations derive significantly greater relative benefits per 1mmol/L LDL-C lowering. Conversely, higher-risk populations derive less benefit per 1mmol/L LDL-C lowering and hence require greater absolute LDL-C reductions to achieve comparable relative treatment benefits. PROSPERO (CRD420251155320)

**Figure:**
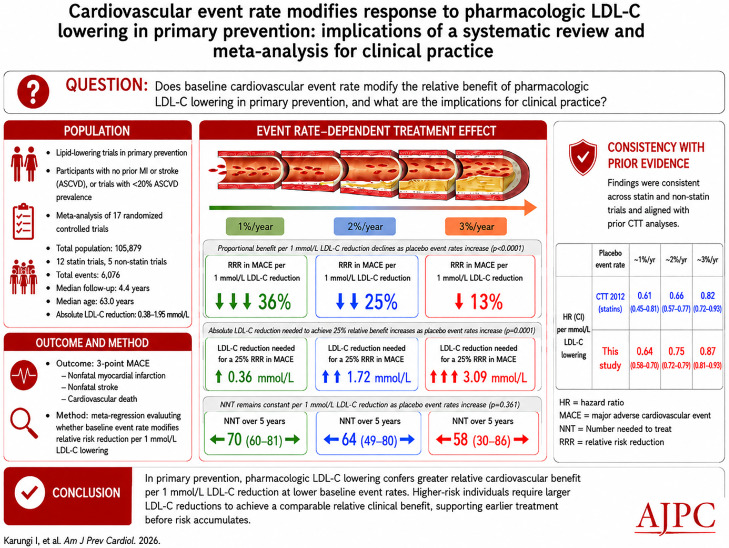
**Central Illustration:** Relative cardiovascular benefit per mmol/L LDL-C lowering is greater at lower baseline risk.

## 1 Introduction

Atherosclerotic cardiovascular disease (ASCVD) is the leading global cause of morbidity and mortality, affecting nearly half a billion individuals and contributing to >19 million deaths annually [1,2]. Its clinical manifestation, most notably as myocardial infarction or stroke, represents the end-stage of a largely silent, decades-long disease process that often begins in childhood. Although the fatty streak, the hallmark of early atherosclerosis [3], begins with the retention of cholesterol-containing particles in the artery wall, mostly from the cholesterol contained in low-density lipoproteins (LDL-C) [4,5], cardiovascular disease is multifactorial and has a polygenic aetiology [6,7]. Risk factors for ASCVD such as hypertension, diabetes, and smoking result in arterial wall injury, resulting in greater retention of cholesterol-containing lipoproteins at lower circulating plasma lipid levels [8,9]. Hence, the relative risk reduction (RRR) per 1mmol/L absolute lowering is relatively constant irrespective of the drivers of risk, whether that be diabetes, hypertension, smoking, low HDL-C or high LDL-C [10]. However, the absolute benefit from per 1mmol/L LDL-C lowering varies by baseline risk and is greater when baseline risk is higher [10].

Unlike cancer management, where the focus is on detection and management of cancer-risk early [11], current prevention strategies for ASCVD adopt a risk-based approach, prioritizing more intensive therapy for those with the highest baseline-risk [12]. While pragmatic for cost-effectiveness, populations targeted by these approaches are more likely to have a greater atherosclerotic plaque burden [13] and more advanced atherosclerotic disease [14]. These plaques are less susceptible to stabilization or regression [15] from single first-line therapies such as statins alone [16,17] resulting in the need for adjunctive lipid-lowering therapies [[18], [19], [20]].

Current recommendations are informed by randomized controlled trials (RCTs), which, despite demonstrating a ∼25 % risk reduction in primary prevention [21,22], typically span only 3–5 years and, in low-risk populations, provide little impetus for the consideration of the potential longer-term health gains which might be afforded by initiating preventive therapies at lower-risk [5], potentially delaying the need for initiation of add-on lipid-lowering-therapies. By contrast, Mendelian randomization captures long-term exposure and has shown a 50–60 % proportional reduction in risk of cardiovascular events per 1mmol/L LDL-C difference [5]. However, its non-interventional design cannot replace RCTs [23]. Moreover, whether the benefits observed in human genetic studies are derived solely from cumulative exposure to lower LDL-C or from plausibly the benefits of LDL-C lowering initiated against an absence of pre-existing atherosclerosis from birth or both cannot be distinguished [5,23]. This limits its direct applicability to guideline recommendations. Trial data have shown that LDL-C lowering is beneficial even at annualized risk of 0.3 % per year [24] and both major global guidelines recommend that LDL-C lowering should be considered even among those with a 10-year risk in the 2–3 % range [12,25]. That said, modest short-term absolute benefits in lower-risk individuals may, for some, not justify the potential longer-term benefits from treatment initiation at lower-risk levels [26].

If randomized controlled trial (RCT) evidence demonstrated that a given unit of LDL-C lowering provided a greater proportional risk reduction at lower event rates [27], it could provide a rationale for physicians and patients to consider initiation of LLTs in lower-risk primary prevention settings. Our study aimed to conduct a systematic review and analysis of available RCT data to i) evaluate how RRR for major adverse cardiovascular events per mmol/L LDL-C lowering varies by placebo event rate among populations with no or low prevalence of established ASCVD and ii) estimate the magnitude of LDL-C reduction required to achieve a 25 % RRR at varying risk thresholds.

## 2 Methods

This systematic review, prospectively registered on PROSPERO (CRD420251155320) and reported according to the Preferred Reporting Items for Systematic Reviews (**Supplemental Table 1**) guidelines [28,29], identified, evaluated, and synthesized evidence on the cardiovascular risk-reduction effects of statin and non-statin therapies in populations with low or no prevalence of established ASCVD, across varying baseline cardiovascular risk. Relative risk reductions per 1mmol/L across a range of event rates from the Cholesterol Treatment Trialists’ Collaboration 2012 (CTT-2012) were used to contextualize findings. A trial-level meta-analytic framework pooled randomized evidence and assessed effect modification of LDL-C–associated risk reduction by baseline cardiovascular risk (proxied by placebo event rates).

### 2.1 Eligibility criteria

Studies were selected based on predefined inclusion and exclusion criteria (**Supplemental Table 2**). Eligible studies were completed RCTs (and one ongoing RCT for exploratory analyses only) that enrolled participants with low or no prevalence of clinically manifest ASCVD, where ASCVD was defined as coronary artery disease (including myocardial infarction or coronary revascularization), cerebral vascular disease (including stroke or transient ischaemic attack), or other atherosclerotic complications. Trials that enrolled mixed populations (participants with or without established ASCVD) were included only if the primary prevention subgroup was reported separately, or if participants with ASCVD comprised ≤20 % of the study population [22]. Only RCTs that evaluated lipid-lowering therapy (LLT) compared with placebo or usual care were included. Additionally, eligible trials were required to report either 3-point major adverse cardiovascular events (3P-MACE), defined as the composite of non-fatal myocardial infarction, non-fatal stroke, and cardiovascular death, or its individual components. Trials were excluded if they enrolled fewer than 1000 participants, had a median follow-up of <1 year, or were still ongoing without published baseline characteristics as of March 2026.

### 2.2 Data sources and search strategy

A comprehensive systematic search was conducted in Embase (Ovid), Medline (Ovid), and the Cochrane Central Register of Controlled Trials (CENTRAL) via the Cochrane Library. Structured search strategies were developed and conducted for each database from inception to March 30, 2026. To ensure comprehensive retrieval, controlled vocabulary terms (Medical Subject Headings and Emtree terms) were combined with free-text keywords, as detailed in **Supplemental Tables 3a, 3b, and 3c**.

### 2.3 Data extraction

Two reviewers (CATS and IK) independently screened titles and abstracts using Covidence. Full-text articles were assessed by three reviewers (JB, CATS, IK) against predefined criteria, with discrepancies resolved by consensus and an additional reviewer (KKR, JB, CATS, IK). Data were extracted independently by two reviewers (CATS and IK) using a standardized extraction form capturing study, participant, intervention, and study outcome characteristics (**Supplemental Table 4**).

### 2.4 Quality assessment

Two reviewers (CATS and IK) independently assessed risk of bias using the Cochrane Risk of Bias tool 2.0 [30], resolving discrepancies through discussions.

### 2.5 Study outcomes

The primary outcome was 3P-MACE, defined as the composite of non-fatal myocardial infarction, non-fatal stroke, and cardiovascular death. For trials that did not directly report 3P-MACE, the composite was estimated using inverse-variance weighting of the individual 3P-MACE components.

### 2.6 Data analysis

#### 2.6.1 Trial-level characteristics and derived variables

For each completed trial, the mean absolute LDL-C reduction from the intervention was calculated as the average of the between-group LDL-C change from baseline at one year and at study completion. The annualized placebo event rate was calculated by dividing the 3P-MACE rate for the control arm of each trial by the median follow-up duration; when 3P-MACE was not reported directly, the annualized placebo event rate was approximated as the sum of the annualized rates of the individual 3P-MACE components. Relative risk reduction per mmol/L LDL-C lowering was used to characterize proportional treatment effects across trial populations. Although absolute risk reduction and number needed to treat are central to clinical decision-making, these measures scale directly with baseline event rates and were therefore not the primary focus of this analysis.

#### 2.6.2 Relationship between baseline risk and RRR using CTT-2012 estimates

To contextualize the relationship between baseline cardiovascular risk and LDL-C–associated risk reduction, published summary data from the CTT-2012 meta-analysis for participants without established cardiovascular disease were plotted [21] (assuming a log-linear relationship between RRR and absolute LDL-C lowering). Linear regression was used to generate best-fit regression lines for the relative risk reduction (RRR) per 1 mmol/L LDL-C lowering across annual placebo event rates, which represented 5-year baseline risk strata in CTT-2012. In CTT-2012, observed events were major vascular events defined as major coronary events (ie, non-fatal myocardial infarction or coronary death), strokes, or coronary revascularizations. From the fitted regression equation, the LDL-C reduction required to achieve a 25 % RRR was estimated for each placebo event rate category in CTT-2012.

Trials not included in CTT-2012 were plotted using their observed RRR and 95 % CI and observed LDL-C difference against the slopes of the CTT-2012 estimates to assess whether these trials followed a similar pattern. Two trials [31,32] were plotted using a CTT endpoint (as this was reported), whereas the remaining trials were plotted using their 3P-MACE endpoints.

#### 2.6.3 Relationship between baseline risk and RRR using effect modification meta-analysis

Trial-level data of all available RCTs were included in a mixed-effects meta-regression to assess whether the annualized placebo event rate modified the RRR for 3P-MACE. To enable comparison across trials with differing magnitudes of LDL-C lowering, treatment effects were standardized by expressing the 3P-MACE hazard ratio (HR) per 1 mmol/L reduction in LDL-C. For each trial, the reported HR for 3P-MACE was log-transformed and divided by the trial’s mean absolute LDL-C reduction between treatment groups. Then the exponent of the resulting coefficient was used to obtain the standardized HR per 1 mmol/L LDL-C reduction. The corresponding RRR was obtained as 1-standardized HR.

For each trial, the number needed to treat (NNT) to prevent one 3P-MACE event over 5 years per 1 mmol/L reduction in LDL-C was estimated as the reciprocal of the standardized 5-year absolute risk reduction. The relationship between NNT and baseline cardiovascular risk was assessed using weighted meta-regression.

To assess whether RRR per 1 mmol/L LDL-C reduction differed between trials independently of baseline risk, a mixed effects meta-regression model was conducted by drug class while adjusting for placebo event rate.

Sensitivity analyses were conducted for trials enrolling participants with 0 % ASCVD prevalence and for individual components of 3P-MACE (non-fatal myocardial infarction, non-fatal stroke, and cardiovascular death). For each component, treatment effects were standardized per 1 mmol/L LDL-C reduction and analyzed using the same meta-regression framework to assess effect modification by baseline risk. Further details for subgroup analyses are provided in the supplementary methods.

#### 2.6.4 Estimation of LDL-C reduction required for a 25 % risk reduction

The target RRR of 25 % was selected a priori based on the aggregate treatment effect observed in the population without established ASCVD of the CTT-2012 meta-analysis, reflecting the cumulative cardiovascular benefit per mmol/L LDL-C lowering [21]. We estimated the LDL-C reduction corresponding to a 25 % RRR in 3P-MACE by dividing each trial’s mean absolute LDL-C reduction by its observed 3P-MACE RRR and multiplying by 25. These trial-level estimates were plotted against the placebo event rate, and a best-fit line was obtained using linear regression.

### 2.7 Ethical considerations

This study was based on secondary analysis of publicly available data from published studies; hence, separate ethical approval was not required.

## 3 Results

Systematic database searches identified 2183 records (Embase: 1162, MEDLINE: 611, and CENTRAL: 410); after duplicate removal, 1711 were screened, of which 68 underwent full-text evaluation. Eighteen RCTs met inclusion criteria (**Supplemental Figure 1**) [24,[31], [32], [33], [34], [35], [36], [37], [38], [39], [40], [41], [42], [43], [44], [45], [46], [47]], of which 17 were completed [24,[31], [32], [33], [34], [35], [36], [37], [38], [39], [40], [41], [42], [43], [44], [45], [46]], while one remains ongoing with available baseline participant data [47].

### 3.1 Characteristics and outcomes of completed RCTs

The 17 completed trials enrolled 105,879 participants (median follow-up duration of 4.4 years), reporting 6076 3P-MACE events. Twelve trials evaluated statin therapy [24,[33], [34], [35], [36], [37], [38], [39], [40],[43], [44], [45]], two trials evaluated statins in combination with ezetimibe [41,42], and three evaluated non-statin therapies: ezetimibe [46], bempedoic acid [31], and a Proprotein Convertase Subtilisin/Kexin Type 9 (PCSK9) inhibitor [32].

Trial populations were diverse, encompassing individuals with hypertension [36], diabetes [32,37,39], chronic kidney disease [42], Human Immunodeficiency Virus [24] and rheumatoid arthritis [45]. Age distributions extended to elderly individuals [35,46], with the highest mean age of 80.6 years [46]. Gender representation was also notably diverse, with the proportion of male participants ranging from 25 % [46] to 100 % [33]. Although most trials enrolled individuals without clinically manifest ASCVD [24,31,32,34,35,[37], [38], [39], [40], [41],[43], [44], [45]], four studies had a small proportion of patients with established ASCVD, reaching up to approximately 15 % [33,36,42,46]. Use of background LLT was low (<30 %) across trials, with one notable exception where >90 % of participants were on LLT at baseline and during follow-up [32]. Further details on trial characteristics are provided in Tables 1 **and Supplemental Table 5.**

**Table 1:** Design, features, and population characteristics of completed statin and non-statin trials included in this study.

Trial	Total N	Population	Follow-up (years)^a^	Age (years)^b^	Female, n ( %)	Baseline LDL-C (mmol/L)^c^	BMI (kg/m²)^d^	SBP (mmHg)^e^	Hypertension, n ( %)	DM, n ( %)	Current smoker, n ( %)^f^	Prior ASCVD, n ( %)
**TRIALS REPORTED IN CTT-2012 META-ANALYSIS**												
WOSCOPS [33]	6595	45–64-year-old men, LDL-*C* ≥ 4.0 mmol/L	4.9	55.2 ± 5.5	0 (0.0)	4.97	26.0 ± 3.2	135.5 ± 17.5	1037 (15.7)	76 (1.2)	2905 (44.0)	531 (8.1)
AFCAPS/TeXCAPS [34]	6605	Adults, LDL-C 3.4–7.9mmol/L, HDL<1.2mmol/L	5.2	58.0 ± 7.0	997 (15.1)	3.89	26.8 ± 3.4	138.0 ± 17.0	1448 (21.9)	155 (2.3)	818 (12.4)	0 (0.0)
PROSPER^g^^,^^h^ [35]	3239	Elderly, 70–82 years with high ASCVD risk	3.8	75.1 ± 3.3	1894 (58.5)	3.80	26.9 ± 4.3	156.8 ± 21.5	1621 (50.0)	397 (12.3)	1081 (33.4)	0 (0.0)
ASCOT-LLA [36]	10,305	Hypertensive (40–79 years) TC ≤6·5 mmol/L	3.3	63.1 ± 8.5	1942 (18.8)	3.44	28.6 ± 4.7	164.2 ± 17.8	10,305 (100.0)	2532 (24.6)	3374 (32.7)	1514 (14.7)
CARDS [37]	2838	Type 2 DM (40–75 years), LDL-*C* ≤ 4·1 mmol/L	3.9	61.6 ± 8.2	909 (32.0)	3.04	28.7 ± 3.6	144.0 ± 16.0	2377 (83.8)	2838 (100.0)	631 (22.2)	0 (0.0)
MEGA [38]	7832	40–70 years, TC 5·69–6·98 mmol/L	5.3	58.3 ± 7.2	5356 (68.4)	4.05	23.8 ± 3.0	132.2 ± 16.8	3277 (41.8)	1632 (20.8)	1614 (20.6)	0 (0.0)
ASPEN^g^ [39]	1905	Type 2 DM (40–75 years), LDL-*C* ≤ 4.1 mmol/L	4	60.5 ± 8.3	718 (37.7)	2.95	28.9 ± 3.7	133.0 ± 16.8	540 (28.3)	1905 (100.0)	251 (13.2)	0 (0.0)
JUPITER [40]	17,802	Adults, LDL-*C* ≤ 3.4 mmol/L hs-CRP ≥ 2.0mg/L	1.9	66.0 (60.0–71.0)	6801 (38.2)	2.79	28.4 (25.3–32.0)	134.0 (124.0–145.0)	NR	NR	2820 (15.8)	0 (0.0)
**TRIALS NOT REPORTED IN CTT-2012 META-ANALYSIS**												
SEAS [41]	1873	Asymptomatic mild-moderate AS (≥ 65 years)	4.4	67.6 ± 9.5	723 (38.6)	3.62	26.9 ± 4.3	144.8 ± 20.2	965 (51.5)	NR	360 (19.2)	0 (0.0)
SHARP [42]	9270	Patients aged 40 years and older with CKD	4.9	62.0 ± 12.0	3470 (37.4)	2.77	27.1 ± 5.7	139.0 ± 22.0	NR	2094 (22.6)	1234 (13.3)	1393 (15.0)
HOPE-3 [43]	12,705	Men ≥ 55 years or women ≥ 65 years at ASCVD risk	5.6	65.8 ± 6.4	3435 (27.0)	3.31	27.1 ± 4.8	138.0 ± 14.8	4814 (37.9)	731 (5.8)	3524 (27.7)	0 (0.0)
ALLHAT-LLT^g^ [44]	2867	Individuals ≥65 years with stage 1 or 2 hypertension	5.6	71.3 ± 5.2	1415 (49.4)	3.82	29.5 ± 5.8	147.5 ± 15.4	2867 (100.0)	1463 (51.0)	641 (22.4)	0 (0.0)
TRACE-RA [45]	3002	Rheumatoid arthritis in >50 years old individuals	2.5	61.0 ± 8.4	2227 (74.2)	3.20	26.6 (23.9–30.1)	NR	667 (22.2)	NR	469 (15.6)	0 (0.0)
EWTOPIA 75 [46]	3411	Individuals ≥75 years, with LDL-*C* ≥ 3.6 mmol/L	4.1	80.6 ± 4.7	2539 (74.4)	4.19	23.6 ± 3.6	136.4 ± 15.9	3029 (88.8)	867 (25.4)	171 (5.0)	344 (10.1)
REPRIEVE [24]	7769	Patients aged 40–75 years with HIV	5.1	50.0 (45.0–55.0)	2419 (31.1)	2.79	25.8 (22.8–29.4)	NR	2774 (35.7)	37 (0.5)	1934 (24.9)	0 (0.0)
CLEAR Outcomes^g^ [31]	4206	18–85-year-old individuals, LDL-*C* ≥ 2.6mmol/L	3.3	68.0 ± 6.8	2481 (59.0)	3.69	30.3 ± 5.4	135.8 ± 13.7	3707 (88.1)	2781 (66.1)	NR	0 (0.0)
VESALIUS-CV^g^ [32]	3655	Men ≥ 50–79 or women ≥ 55–79, LDL-*C* ≥ 2.3 mmol/L	4.8	65.0 (60.0–70.0)	5214 (42.5)	3.13	31.4 (28.0–35.5)	NR	3299 (88.3)	3650 (99.9)	1009 (27.6)	0 (0.0)
**Overall** (**weighted**)^i^	**105,879**		**4.4**	**63.0±10.0**	**39,392** (**37.2**)	**3.40**	**27.4±4.9**	**140.7±19.7**	**42,657** (**54.1**)	**21,158** (**25.4**)	**22,836** (**22.5**)	**3782** (**3.6**)

a Age reported as median (Q25-Q75) in JUPITER, REPRIEVE, and VESALIUS-CV; otherwise, all other ages are mean ± SD.

b Follow-up duration reported as mean in WOSCOPS, AFCAPS/TeXCAPS, PROSPER, and MEGA; otherwise, median in all other trials.

c Baseline LDL-C reported as median in JUPITER, TRACE-RA, REPRIEVE, and VESALIUS-CV. To convert LDL-C from mmol/L to mg/dL, multiply by 38.67.

d BMI reported as median in JUPITER, TRACE-RA, REPRIEVE, and VESALIUS-CV; otherwise mean in all other trials.

e SBP reported as median in JUPITER and mean in all other trials.

f Smoking status reported as “current smoker” in all trials except in MEGA, where it is reported as “current/former smoker..”

g Primary prevention cohort results (ASCVD prevalence 0 %) from mixed population trials (PROSPER, ASPEN, ALLHAT-LLT, CLEAR Outcomes and VESALIUS-CV) were included in this analysis.

h All Baseline characteristics for PROSPER, except for LDL-C, were calculated from counts reported by Ian Ford et al. [48]. The baseline LDL-C for PROSPER was reported by Chris J Packard et al. [49].

I Denominator based on trials reporting the given variable. AFCAPS/TeXCAPS; Air Force/Texas Coronary Atherosclerosis Prevention Study, ALLHAT-LLT; Antihypertensive and Lipid-Lowering Treatment to Prevent Heart Attack Trial-Lipid Lowering Trial, ASCOT-LLA; Anglo-Scandinavian Cardiac Outcomes Trial-Lipid Lowering Arm, ASCVD; atherosclerotic cardiovascular disease, ASPEN; Atorvastatin Study for Prevention of Coronary Heart Disease Endpoints in Non-Insulin-Dependent Diabetes Mellitus, CARDS; Collaborative Atorvastatin Diabetes Study, CHD; coronary heart disease, CLEAR Outcomes; Cholesterol Lowering via Bempedoic Acid, an ACL-Inhibiting Regimen Outcomes, CVD; cardiovascular disease, DM; diabetes mellitus, EWTOPIA 75; Ezetimibe Lipid-Lowering Trial On Prevention of Atherosclerosis in 75 or Older; HIV; human immunodeficiency virus, HOPE-3; Heart Outcomes Prevention Evaluation-3, hs-CRP; high sensitivity C-reactive protein, JUPITER; Justification for the Use of Statins in Prevention: an Intervention Trial Evaluating Rosuvastatin, LDL-C; low density lipoprotein cholesterol, MEGA; Management of Elevated Cholesterol in the Primary Prevention Group of Adult Japanese, MI; myocardial infarction, NCEP; national cholesterol education program, PP; Primary prevention, PROSPER; PROspective Study of Pravastatin in the Elderly at Risk, REPRIEVE; Randomized Trial to Prevent Vascular Events in HIV, SEAS; Simvastatin and Ezetimibe in Aortic Stenosis, SHARP; Study of Heart and Renal Protection, TRACE-RA; Trial of Atorvastatin for the Primary Prevention of Cardiovascular Events in Patients with Rheumatoid Arthritis, VESALIUS-CV; The Effect of Evolocumab in Patients at High Cardiovascular Risk without Prior Myocardial Infarction or Stroke, WOSCOPS; West of Scotland Coronary Prevention Study.

Key outcomes, including changes in LDL-C, cardiovascular outcomes for 3P-MACE, and annualized placebo event rates for the eligible trials, are shown in Table 2. The mean absolute LDL-C reduction between treatment groups across the trials ranged from 0.38 mmol/L [31,38] to 1.95 mmol/L [41]. Baseline risk, approximated by the annualized placebo event rate for 3P-MACE, varied across trials. The lowest annualized placebo event rates (<1 %/year) were observed in three trials (0.52 %, 0.54 %, and 0.65 %), which enrolled slightly younger populations, with a median age below 60 years [24,34,38]. On the other hand, the highest placebo event rates that exceeded 3 %/year were observed in two trials involving elderly patients [35] or those with significant renal impairment [42]. Irrespective of age, trials with a placebo event rate greater than 1 % had more male participants, current smokers, and a higher prevalence of hypertension and diabetes, as shown in **Supplemental Table 6.**

**Table 2:** Key outcomes of completed statin and non-statin primary prevention trials included in the current analysis.

Trial	Lipid Outcomes					Cardiovascular outcomes								
	LDL-C at 1 year, mmol/L	LDL-C at study completion in intervention arm, mmol/L	Absolute LDL-C change between arms at 1 year, mmol/L	Absolute LDL-C change between arms at study completion, mmol/L	Mean absolute LDL-C reduction, mmol/L	Study arm	Participants (n)	Non-fatal MI	Non-fatal stroke	CV death	Total event count	HR (95 % CI) for 3P-MACE^a^ (unstandardised)	HR (95 % CI) for 3P-MACE (standardised)^b^	Annual placebo event rate ( %)
**TRIALS REPORTED IN CTT-2012 META-ANALYSIS**														
WOSCOPS [33]	3.85	4.11	1.12	0.86	0.99	Placebo	3293	246	47	73	366	0.74 (0.63–0.87)	0.74 (0.63, 0.87)	2.27
						Pravastatin (40 mg)	3302	182	40	50	272			
AFCAPS/TeXCAPS^c^ [34]	2.96	NR	0.92	NR	0.92	Placebo	3301	95	17	25	137	0.63 (0.47–0.85)	0.61 (0.44, 0.84)	0.65
						Lovastatin (20–40 mg)	3304	57	14	17	88			
PROSPER [35] d^,^^f^^,^^g^	NR	2.50	NR	1.30	1.30	Placebo	1654	NR	NR	NR	200	0.94 (0.77–1.16)	0.96 (0.82, 1.12)	3.78
						Pravastatin (40 mg)	1585	NR	NR	NR	181			
ASCOT-LLA [36] c	2.25	2.32	1.20	1.95	1.08	Placebo	5137	154	121	82	357	0.68 (0.57–0.82)	0.70 (0.59, 0.83)	1.62
						Atorvastatin (10 mg)	5168	100	89	74	263			
CARDS [37]	1.86	2.11	1.24	1.01	1.13	Placebo	1410	41	30	29	100	0.63 (0.46–0.87)	0.67 (0.50, 0.88)	1.82
						Atorvastatin (10 mg)	1428	25	20	19	64			
MEGA [38]	3.30	3.17	0.67	0.50	0.59	NCEP step 1 diet	3966	30	62	18	110	0.72 (0.54–0.96)	0.57 (0.35, 0.93)	0.52
						NCEP step 1 diet + Pravastatin (10–20 mg)	3866	16	50	11	77			
ASPEN [39] c^,^^f^	NR	2.05	NR	0.90	0.90	Placebo	946	34	29	19	82	0.95 (0.70–1.29)	0.85 (0.57, 1.27)	1.66
						Atorvastatin (10 mg)	959	28	27	24	79			
JUPITER [40]	1.42	1.42	1.42	1.39	1.41	Placebo	8901	62	58	37	157	0.53 (0.41–0.69)	0.64 (0.53, 0.77)	0.93
						Rosuvastatin (20 mg)	8901	22	30	31	83			
**TRIALS NOT REPORTED IN CTT-2012 META-ANALYSIS**														
SEAS [41]	NR	1.67	NR	1.95	1.95	Placebo	929	26	29	56	111	0.86 (0.66–1.13)	0.93 (0.80, 1.06)	2.72
						Simvastatin (40 mg) + ezetimibe (10 mg)	944	17	33	47	97			
SHARP [42]	1.69	1.93	1.09	0.77	0.93	Placebo	4620	159	133	388	680	0.88 ( 0.79–0.98)	0.87 (0.78, 0.98)	3.00
						Simvastatin (20 mg) + ezetimibe (10 mg)	4650	134	108	361	603			
HOPE-3 [43] e	2.29	2.25	1.02	0.76	0.89	Placebo	6344	69	99	171	339	0.76 (0.64–0.91)	0.73 (0.60, 0.90)	0.86
						Rosuvastatin (10 mg)	6361	45	70	154	269			
ALLHAT-LLT [44] f^,^^g^	2.91	2.82	0.62	0.51	0.57	Usual care + NCEP step 1 diet	1400	78	52	87	217	0.93 (0.77–1.13)	0.88 (0.63, 1.23)	2.77
						NCEP step 1 diet + Pravastatin (40 mg)	1467	58	53	101	212			
TRACE-RA [45]	NR	2.21	NR	0.77	0.77	Placebo	1498	20	12	6	38	0.60 (0.36–1.01)	0.52 (0.26, 1.02)	1.01
						Atorvastatin (40 mg)	1504	11	6	6	23			
EWTOPIA 75 [46]	3.26	3.11	0.47	0.29	0.38	Dietary counseling	1695	16	54	45	115	0.72 (0.55–0.96)	0.61 (0.40, 0.93)	1.65
						Dietary counseling + ezetimibe (10 mg)	1716	8	47	29	84			
REPRIEVE [24]	1.99	2.07	0.80	0.60	0.70	Placebo	3881	47	44	16	107	0.63 (0.46–0.85)	0.51 (0.33 0.79)	0.54
						Pitavastatin (4 mg)	3888	26	29	12	67			
CLEAR Outcomes [31] e^,^^f^^,^^h^	2.92	2.87	0.60	0.32	0.60	Placebo	2106	44	33	65	142	0.64 (0.48–0.84)	0.48 (0.30, 0.76)	1.93
						Bempedoic acid (180 mg)	2100	28	26	37	91			
VESALIUS-CV [32] e^,^^f^	1.34	1.14	1.53	1.58	1.56	Placebo	1806	53	40	63	156	0.68 (0.52–0.88)	0.78 (0.66, 0.92)	1.58
						Evolocumab (140 mg)	1846	37	28	44	109			

a 3P-MACE endpoint for this study was defined as non-fatal MI, non-fatal stroke, and cardiovascular death.

b 3P-MACE standardized per 1 mmol/L LDL-C reduction obtained by dividing the log-transformed unstandardized HR by the average absolute LDL-C reduction achieved in each trial and transforming it back to the original HR format by exponentiation.

c Counts for MI and stroke were reported as combined counts for fatal and non-fatal events in AFCAPS/TeXCAPS, ASCOT-LLA, and ASPEN. For these trials, the 3P-MACE endpoint and the annual placebo event rate were calculated from counts of fatal and non-fatal events, excluding CV death.

d The composite total event count and HR reported in PROSPER comprised non-fatal MI, fatal/non-fatal stroke, and CHD death.

e The Composite total event count and HR for 3P-MACE reported in HOPE-3, CLEAR Outcomes, and VESALIUS-CV were used in all analyses, not those derived from summing individual components.

f Primary prevention cohort results (ASCVD prevalence 0 %) from mixed population trials (PROSPER, ASPEN, ALLHAT-LLT, CLEAR Outcomes and VESALIUS-CV) were included in this analysis.

g LDL-C change from baseline for PROSPER (at 3 months) was reported by Chris J Packard et al. [49]. The earliest LDL-C change from baseline reported in ALLHAT-LLT is at 2 years.

h For CLEAR Outcomes, LDL-C absolute change between-groups at 1 year was reported by Lincoff et al. [51]. Time-averaged between-group change in LDL-C, over study follow-up, used as reported by Nissen et al. [31]. Nonfatal MI and nonfatal stroke counts are from data on file, reported by Nissen et al. [50] To convert mmol/L to mg/dL, multiply by 38.67 AFCAPS/TeXCAPS; Air Force/Texas Coronary Atherosclerosis Prevention Study, ALLHAT-LLT; Antihypertensive and Lipid-Lowering Treatment to Prevent Heart Attack Trial-Lipid Lowering Trial, ASCOT-LLA; Anglo-Scandinavian Cardiac Outcomes Trial-Lipid Lowering Arm, ASPEN; Atorvastatin Study for Prevention of Coronary Heart Disease Endpoints in Non-Insulin-Dependent Diabetes Mellitus, CARDS; Collaborative Atorvastatin Diabetes Study, CLEAR Outcomes; Cholesterol Lowering via Bempedoic Acid, an ACL-Inhibiting Regimen Outcomes, CV; cardiovascular, EWTOPIA 75; Ezetimibe Lipid-Lowering Trial On Prevention of Atherosclerosis in 75 or Older; HOPE-3; Heart Outcomes Prevention Evaluation-3, HR; hazard ratio, JUPITER; Justification for the Use of Statins in Prevention: an Intervention Trial Evaluating Rosuvastatin, LDL-C; low density lipoprotein cholesterol, MEGA; Management of Elevated Cholesterol in the Primary Prevention Group of Adult Japanese, MI; myocardial infarction, NA; not available, PROSPER; PROspective Study of Pravastatin in the Elderly at Risk, REPRIEVE; Randomized Trial to Prevent Vascular Events in HIV, RRR; relative risk reduction, SEAS; Simvastatin and Ezetimibe in Aortic Stenosis, SHARP; Study of Heart and Renal Protection, TRACE-RA; Trial of Atorvastatin for the Primary Prevention of Cardiovascular Events in Patients with Rheumatoid Arthritis, VESALIUS-CV; The Effect of Evolocumab in Patients at High Cardiovascular Risk without Prior Myocardial Infarction or Stroke, WOSCOPS; West of Scotland Coronary Prevention Study, 3P-MACE; 3-point major adverse cardiovascular event.

### 3.2 Analyses using estimates from the cholesterol treatment trialists’ collaboration (CTT-2012)

**Fig. 1:**
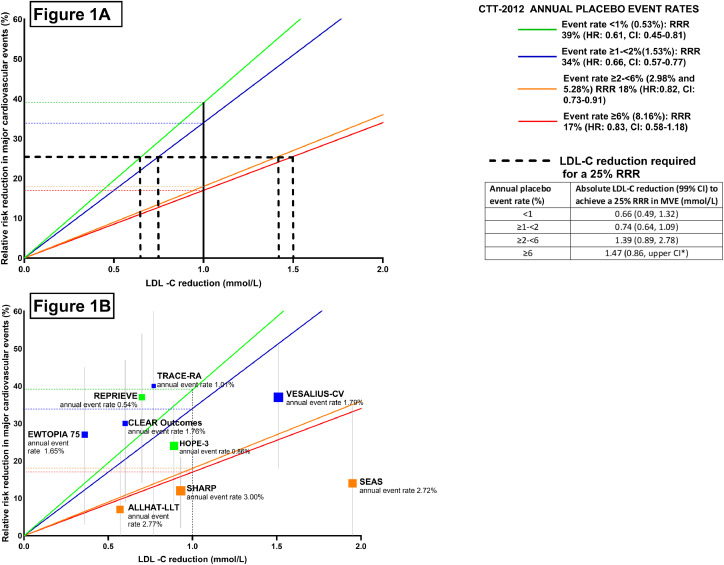
RRR by risk threshold (event rates) using CTT-2012 estimates for major cardiovascular events (**1A**) and alongside the CTT 2012 framework with trials not included in the CTT-2012 report (**1B**). **1A:** RRR per 1 mmol/L LDL-C lowering across annual placebo event rates in CTT-2012 (using between group LDL-C differences at 1year). **1B:** RRR for trials not included in CTT-2012 plotted using the mean (during the overall trial) between-group differences in the absolute LDL-C reductions for each treatment group within each trial. Regression lines represent the RRR per 1 mmol/L LDL-C lowering across annualized placebo event rates, corresponding to 5-year baseline cardiovascular risk categories among primary prevention participants in the CTT-2012. Colors indicate placebo event rate categories. Squares represent individual trials, with size proportional to study weight. RRR attenuates with increasing annual placebo event rates in CTT-2012 meta-analysis and in trials not included in CTT-2012. *Upper CI for LDL-C corresponding to placebo event rate ≥6 is undefined as the HR (99 % CI) for this stratum crossed the null. *CLEAR Outcomes and VESALIUS-CV estimates correspond to the unstandardized CTT-2012 endpoint reported by Lincoff et al [51] and Marston [32], respectively. Estimates for all other trials are for unstandardized 3P-MACE. ALLHAT-LLT; antihypertensive and lipid-lowering treatment to prevent heart attack trial–lipid-lowering trial, CLEAR Outcomes; cholesterol lowering via bempedoic acid, an ACL-inhibiting regimen, EWTOPIA 75; ezetimibe lipid-lowering trial on prevention of atherosclerotic cardiovascular disease in 75 or older, HOPE-3; heart outcomes prevention evaluation-3 trial, REPRIEVE; randomized trial to prevent vascular events in HIV, RRR; relative risk reduction; SEAS; simvastatin and ezetimibe in aortic stenosis, SHARP; study of heart and renal protection, TRACE-RA; Trial of Atorvastatin for the Primary Prevention of Cardiovascular Events in Patients with Rheumatoid Arthritis. VESALIUS-CV; The Effect of Evolocumab in Patients at High Cardiovascular Risk without Prior Myocardial Infarction or Stroke.

We used risk-stratified effect estimates from the CTT-2012 primary-prevention subgroup to contextualize our analyses. Eight [[33], [34], [35], [36], [37], [38], [39], [40]] of the 17 completed trials in our study contributed data to the CTT-2012 primary prevention population, and the remaining 9 trials [24,31,32,[41], [42], [43], [44], [45], [46]] were not included in CTT-2012. Overall, among primary prevention participants in CTT-2012, each mmol/L reduction in LDL-C from statin therapy decreased the risk of a MACE by 25 % [21]. The event rate categories and their corresponding RRR, as reported by CTT-2012 and utilized in the present study, are detailed in **Supplemental Table 7.** The relative risk reduction in cardiovascular events varied across the CTT-2012 risk strata. In individuals with an annual placebo event rate <1 %/year, the relative risk of an event was reduced by 39 %, which further decreased to 34 % in those with a placebo event rate of 1-<2 %/year. By contrast, as placebo event rates exceeded 6 %/year, the relative risk reduction per 1 mmol/L LDL-C lowering fell to around 17 % (Fig. 1**A**).

Additionally, the absolute LDL-C required to achieve a 25 % RRR increased with higher baseline risk thresholds. In the lower baseline risk categories (placebo event rates <1 % and ≥1-<2 %/year), a 25 % RRR was achieved with small LDL-C reductions of approximately 0.66 and 0.74 mmol/L, respectively. By contrast, the higher baseline risk categories (placebo event rates ≥2-<6 % and ≥6 %/year) required greater LDL-C reductions of approximately 1.39 mmol/L and 1.47 mmol/L, respectively, to attain the same 25 % RRR (Fig. 1**A**)**.**

The 9 trials not included in the CTT-2012 publication evaluated both statin and non-statin therapies, and only a minority (*N* = 2) enrolled populations with a low prevalence of clinically evident ASCVD [42,46]. The majority of the trials were closely aligned with the CTT estimates, with their confidence intervals crossing the CTT-2012 regression lines, which corresponded to the respective annualized placebo event rate for a given trial (Fig. 1**B**). This pattern was consistent when the trial estimates were standardized to a per-1 mmol/L LDL-C reduction (**Supplemental Figure 2**) and when analyses were restricted to trials with no (0 %) clinically evident ASCVD (**Supplemental Figure 3**)**.** A similar trend for the 9 trials not included in CTT-2012 was noted, with the RRR attenuating as risk increased, consistent with the CTT-2012 data. The trials with the highest annualized event rates (2.72 %, 2.77 %, and 3.00 %) had the lowest per mmol/L standardized RRRs (7–13 %) [41,42,44], whereas the trial with the lowest event rate (0.54 %) was among those with high standardized per mmol/L RRR (49 %) [24]. One trial (VESALIUS-CV) showed a modest deviation from this pattern; notably, it had ∼90 % background mostly statin LLT use [32].

### 3.3 Analyses of all available trials

**Fig. 2:**
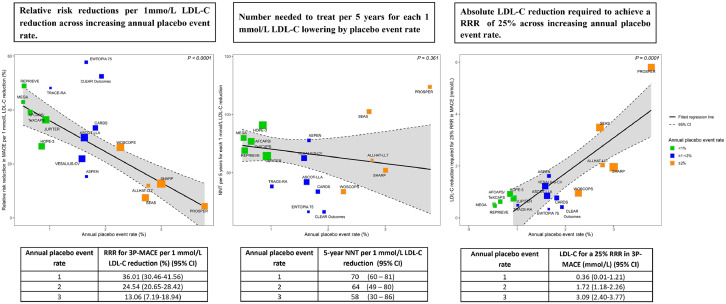
Meta-regression of RRR (y axis) in 3PMACE (**2A**) standardized per 1 mmol/L LDL-C lowering as a function of annualized placebo event rate (x axis); NNT to prevent one 3PMACE over 5 years for each 1 mmol/L LDL-C lowering across the range of annualized placebo event rates (**2B**); absolute LDL-C required to achieve a 25 % RRR (**2C**) across increasing annualized placebo event rate. Squares represent individual trials, with size proportional to study weight. Colors indicate placebo event rate categories. The solid line shows the fitted regression line, and dashed lines indicate the 95 % confidence intervals. RRR per 1mmol/L LDL-C lowering diminished with increasing event rate, whereas 5-year NNT remained constant across increasing event rates; the LDL-C reduction required to achieve a 25 % RRR increased as the placebo event rate increased. 3P-MACE: 3-point major adverse cardiovascular event, AFCAPS/TeXCAPS; Air Force/Texas Coronary Atherosclerosis Prevention Study, ALLHAT-LLT; Antihypertensive and Lipid-Lowering Treatment to Prevent Heart Attack Trial-Lipid Lowering Trial, ASCOT-LLA; Anglo-Scandinavian Cardiac Outcomes Trial-Lipid Lowering Arm, ASPEN; Atorvastatin Study for Prevention of Coronary Heart Disease Endpoints in Non-Insulin-Dependent Diabetes Mellitus, CARDS; Collaborative Atorvastatin Diabetes Study, CLEAR Outcomes; Cholesterol Lowering via Bempedoic Acid, an ACL-Inhibiting Regimen Outcomes, EWTOPIA 75; Ezetimibe Lipid-Lowering Trial On Prevention of Atherosclerosis in 75 or Older; HOPE-3; Heart Outcomes Prevention Evaluation-3, JUPITER; Justification for the Use of Statins in Prevention: an Intervention Trial Evaluating Rosuvastatin, MEGA; Management of Elevated Cholesterol in the Primary Prevention Group of Adult Japanese, NNT; Number needed to treat, PROSPER; PROspective Study of Pravastatin in the Elderly at Risk, REPRIEVE; Randomized Trial to Prevent Vascular Events in HIV, SEAS; Simvastatin and Ezetimibe in Aortic Stenosis, SHARP; Study of Heart and Renal Protection, TRACE-RA; Trial of Atorvastatin for the Primary Prevention of Cardiovascular Events in Patients with Rheumatoid Arthritis, VESALIUS-CV; The Effect of Evolocumab in Patients at High Cardiovascular Risk without Prior Myocardial Infarction or Stroke, WOSCOPS; West of Scotland Coronary Prevention Study.

To further explore these findings, we conducted a meta-regression analysis using trial-level data from all 17 trials of statins and non-statins [24,[31], [32], [33], [34], [35], [36], [37], [38], [39], [40], [41], [42], [43], [44], [45], [46]]. This confirmed that RRR diminished with increasing baseline risk and demonstrated significant effect modification of the 1mmol/L standardized 3P RRR by annualized placebo event rate (p-value: <0.0001), Fig. 2**A.** The meta-regression showed that, for a 1 mmol/L LDL-C reduction, lower-risk populations with an annualized placebo event rate of 1 % derived nearly threefold greater relative benefits (36 % RRR) than those with a placebo event rate of 3 % (13 % RRR). This trend for attenuated benefits at increasing risk thresholds was consistent in sensitivity analyses when restricted to trials enrolling participants with 0 % prevalence of clinically evident ASCVD (**Supplemental Figure 4A**)**,** trials reporting only between-arm LDL-C difference at 1-year (**Supplemental Figure 5**), trials stratified by median duration of follow-up and baseline LDL-C (**Supplemental Figure 6A and 6B**), and with prior observations in CTT-2012 (**Supplemental Table 7 and 8**). Additional sensitivity analyses excluding TRACE-RA (*p* < 0.0001) or SEAS (*p* < 0.0001) showed consistent patterns. Across the 17 trials, when RRR was standardized per 1mmol/L LDL-C lowering, the HR was 0.72 (CI 0.68–0.76), I^2^ 0 %, *p* = 0.311. (**Supplemental Figure 7**)**.** Notably, the number needed to treat over 5 years for each mmol/L LDL-C lowering did not differ significantly by baseline risk (*P* = 0.361) (Fig. 2**B**)**.**

**Fig. 3:**
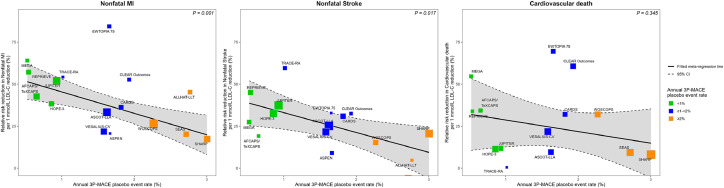
Meta-regression showing standardized RRR per 1 mmol/L LDL-C reduction (y axis) plotted against placebo annualized event rate for individual components of 3P-MACE: Nonfatal MI, Nonfatal stroke, and cardiovascular death. Squares represent individual trials, with size proportional to study weight. Colors indicate placebo event rate categories. The solid line shows the fitted regression line, and dashed lines indicate the 95 % confidence intervals. Trials not reporting a given individual 3P-MACE component were excluded from the corresponding component-specific plot (PROSPER: nonfatal MI, nonfatal stroke and cardiovascular death). Trials with a negative RRR for an individual component were included in the analysis but not displayed in the respective plot (SEAS: nonfatal stroke, ASPEN and ALLHAT-LLT: cardiovascular death). AFCAPS/TeXCAPS; Air Force/Texas Coronary Atherosclerosis Prevention Study, ALLHAT-LLT; Antihypertensive and Lipid-Lowering Treatment to Prevent Heart Attack Trial-Lipid Lowering Trial, ASCOT-LLA; Anglo-Scandinavian Cardiac Outcomes Trial-Lipid Lowering Arm, ASPEN; Atorvastatin Study for Prevention of Coronary Heart Disease Endpoints in Non-Insulin-Dependent Diabetes Mellitus, CARDS; Collaborative Atorvastatin Diabetes Study, CLEAR Outcomes; Cholesterol Lowering via Bempedoic Acid, an ACL-Inhibiting Regimen Outcomes, EWTOPIA 75; Ezetimibe Lipid-Lowering Trial On Prevention of Atherosclerosis in 75 or Older; HOPE-3; Heart Outcomes Prevention Evaluation-3, JUPITER; Justification for the Use of Statins in Prevention: an Intervention Trial Evaluating Rosuvastatin, MEGA; Management of Elevated Cholesterol in the Primary Prevention Group of Adult Japanese, PROSPER; PROspective Study of Pravastatin in the Elderly at Risk, REPRIEVE; Randomized Trial to Prevent Vascular Events in HIV, SEAS; Simvastatin and Ezetimibe in Aortic Stenosis, SHARP; Study of Heart and Renal Protection, TRACE-RA; Trial of Atorvastatin for the Primary Prevention of Cardiovascular Events in Patients with Rheumatoid Arthritis, VESALIUS-CV; The Effect of Evolocumab in Patients at High Cardiovascular Risk without Prior Myocardial Infarction or Stroke, WOSCOPS; West of Scotland Coronary Prevention Study.

Similar to the observations using CTT-2012 data (8 trials), the totality of the evidence (17 trials) suggested that the absolute LDL-C required to achieve a 25 % RRR needed to increase at higher baseline risk thresholds in both the main and sensitivity analyses (Fig. 2**C and Supplemental Figure 4B**). At a low annualized placebo event rates of 1 %, a modest LDL-C absolute reduction of 0.36 mmol/L was required to achieve a 25 % RRR in 3P-MACE. By contrast, as annualized placebo event rates increased to 2 % and 3 %, the LDL-C reduction required to achieve a 25 % RRR in 3P-MACE similarly rose to 1.72 and 3.09 mmol/L, respectively. This trend was similar in the sensitivity analyses restricted to trials enrolling participants without clinically evident ASCVD. The observed relationship between baseline risk and standardized relative risk reduction was consistent when analyses were restricted to non-fatal myocardial infarction and non-fatal stroke, respectively, but not for cardiovascular death (Fig. 3).

### 3.4 Risk of bias

Risk of bias assessments are summarized in **Supplemental Figure 8**. All trials had a low overall risk of bias across all domains, and the certainty of evidence for the 3P-MACE outcome was determined as high overall.

### 3.5 Characteristics and predicted outcomes of the ongoing RCT

One ongoing trial met the eligibility criteria for this study [47]. This trial is evaluating statin therapy in healthy older adults aged 70 years and above (trial characteristics are provided in **Supplemental Table 9A**)**.** The estimated LDL-C reduction for this trial is 1.31 mmol/L, based on an assumed 40 % LDL-C reduction [52] and the starting baseline LDL-C. The predicted RRR for this trial, for the CTT outcome if event rates were high (>6 % per year) would be ∼22 % and conversely would be predicted to increase to ∼48 % if event rates were very-low (< 1 %) (**Supplemental Table 9B**). Visual predictions of RRR under different scenarios vs CTT-2012 are shown in **Supplemental Figure 9A,** together with completed trials, **Supplemental Figure 9B,** and **Supplemental Figure 10**

## 4 Discussion

This systematic review and meta-analysis combined evidence from 17 completed lipid-lowering clinical trials involving 105,879 participants, in populations with no or low prevalence of clinically evident ASCVD. Three key findings emerged (**Central Illustration**). Firstly, trial populations with a lower placebo event rate of 1 %/year derived almost three times the relative benefits as those with event rates exceeding 3 %/year for each 1 mmol/L LDL-C lowering. Secondly, trial populations with lower annualized placebo event rates achieved a 25 % reduction in cardiovascular events with a reduction in LDL-C of <1 mmol/L (approximately 0.4 mmol/L). By comparison, those at higher risk required a six-fold greater reduction in LDL-C (approximately 3 mmol/L) to attain a similar relative benefit. Thirdly, the individual participant data from the 8 trials used in the CTT-2012 meta-analysis showed similar patterns, and while populations varied across trials, the aggregated data from the 9 trials not previously reported in the CTT-2012 were consistent with the CTT-2012 estimates when plotted against their corresponding annual event rates [21].

The findings of the present meta-regression of 17 trials can be contextualized within the established CTT framework. The CTT-2012 meta-analysis demonstrated broadly consistent proportional benefits per 1 mmol/L LDL-C reduction across risk strata in individuals with established ASCVD, in whom plaque burden is likely substantial. However, among individuals without prior cardiovascular events, CTT-2012 reported attenuating proportional benefits as 5-year baseline risk increased [21]. A recent meta-analysis by Kalra et al. included trial data since the CTT-2012 from a total of 14 trials and reported a 30 % RRR per 1mmol/L lowering of LDL-C for a composite 4P-MACE, concluding that this RRR was greater than published benefits in secondary prevention. However, this recent meta-analysis did not quantify how RRR varies continuously across the risk spectrum per unit change in LDL-C, nor estimate the magnitude of LDL-C reduction required to achieve a specific magnitude of relative benefit at different risk thresholds using the harder 3P-MACE endpoint [22]. Our trial-level meta-regression showed the same trend of smaller relative clinical benefits in higher-risk groups, compared to lower-risk groups. This pattern was consistent across therapies and after excluding trials with a low prevalence of ASCVD. Analyses of individual 3P-MACE components followed the same trend, with myocardial infarction and stroke being statistically significant and likely driving the overall composite pattern. A higher RRR in selected primary prevention populations, particularly those with inflammatory conditions [24,40,45], may indicate that populations with heightened inflammation (as manifest by elevated hsCRP), have greater disease modifiability in response to LLTs. However, in trials such as HOPE-3 and ASCOT-LLA, analyses of RRR by tertiles of baseline hsCRP did not show effect modification [43,53]. Alternative explanations for potential trial-level greater RRR could include early trial cessation (JUPITER) [40], which may have inflated the RRR, as well as differences in types of events contributing to the 3P-MACE outcome. The modest deviation observed in VESALIUS-CV might be partially explained by the background LLT use exceeding 90 %, the highest of any included trial, which could attenuate observed placebo event rates for patients already receiving chronic statin therapy versus the untreated state observed in other trials, thereby attenuating the apparent proportional treatment effect from adjunctive LLT.

Imaging studies provide a mechanistic rationale for the observations in the present study. For instance, LLTs reduce cardiovascular events by promoting plaque stabilization and slowing plaque progression [16,17,[54], [55], [56], [57]], yet the extent of plaque modification with LDL-C lowering in a given time window depends on the stage of atherosclerotic disease [58]. In early atherogenesis, first-line LLT, particularly statins, can prevent or stabilize nascent lesions [16,17]. In more advanced disease, however, even high-intensity statins, which reduce LDL-C to 1.6–2.0 mmol/L and arrest plaque progression, are unlikely to substantially regress mature plaques [[55], [56], [57]]. Plaque regression requires more intensive LDL-C reduction to on-treatment levels of ∼0.6–0.9 mmol/L, achievable with, for instance, when adjunctive PCSK9-targeting treatments are added [[18], [19], [20]]. These stage-dependent effects suggest that the proportional clinical benefit of a given LDL-C reduction may vary with the timing of intervention and hence with baseline cardiovascular risk, which, in part, may reflect the underlying plaque burden and/or maturity [14,58]. An alternative explanation for the attenuation of relative benefit at higher baseline risk is competing risks. In higher-risk populations, the probability of dying from non-cardiovascular causes, or from non-atherosclerotic cardiovascular causes, may reduce the observable benefit from LDL-C lowering on atherosclerotic events (3P MACE) [59]. This is particularly relevant at the upper end of the event-rate spectrum included in this analysis, where comorbidity burden was likely greater. Disentangling plaque-stage effects from competing risks would require individual-level data with cause-specific mortality adjustment.

Our observations of LDL-C reduction through diverse pharmacological pathways align with Mendelian randomization evidence showing that cardiovascular benefit is determined by the magnitude of LDL-C lowering rather than the specific therapeutic pathway [5]. For instance, genetic instruments mimicking statins, ezetimibe, and PCSK9 inhibitors demonstrate broadly consistent relative cardiovascular risk reductions of 19–24 % per 1 mmol/L LDL-C lowering [5], consistent with pharmacological comparisons when matched for treatment duration [60,51]. As such, pleotropic effects of these agents minimally contribute to their cardiovascular benefit [5,60,51].

Current guidelines recommend risk-based initiation of lipid-lowering therapy, with more intensive treatment recommended for those with higher absolute risk [61]. This is supported by health-economic reasoning; as these patients derive greater absolute cardiovascular risk reduction and thus have a smaller number needed to treat, assuming constant relative benefits across risk groups [[62], [63], [64]]. First-line treatment is typically statins, which are cost-effective and are now generic [63]. After an index ASCVD event, the risk of further events remains high, and non-statin add-on therapies such as ezetimibe or PCSK9 inhibitors provide additional RRR of _∼_7 %−15 % [[65], [66], [67]]. PCSK9 inhibitors, although historically limited by cost, have shown cost-effectiveness in high-risk subgroups such as patients with diabetes, peripheral arterial disease, or elevated lipoprotein (a) [64,68]. Our findings, however, offer a nuanced perspective on the assumption of the cost-effectiveness advantage of targeting higher-risk primary prevention populations. Given that the relative benefit attenuated with increasing baseline risk, the expected absolute risk reduction was partially offset, such that the number needed to treat to prevent an event per 5 years for each mmol/L reduction in LDL-C was broadly similar across trial risk strata. While this does not negate the role of absolute risk assessment in shared decision-making, it suggests that withholding LDL-C lowering in lower-risk settings may not be financially justified [69], consistent with evidence that even at borderline risk (5–7.4 % 10-year risk), LDL-C lowering can be cost-saving [62].

Our findings further support the life-course strategy whereby modest LDL-C reductions, even when event rates are low, yield clinically relevant proportional cardiovascular benefits. Anchoring on the CTT-2012 benchmark of a ∼25 % proportional reduction in major vascular events per 1 mmol/L LDL-C lowering [21], our trial-level analyses showed that achieving the 25 % risk reduction in higher-risk populations required reductions of 2 mmol/L or more, levels that are often difficult to attain in routine care and often necessitate combination therapy [[65], [66], [67]]. The 2026 ACC/AHA/Multisociety Dyslipidemia guidelines are evolving towards adopting a life-course approach, recommended strong consideration of statin use in persons at least age 30 who have persistent LDL-C values of >4.2 mmol/l (160 mg/dL), a strong family history of premature ASCVD, or an elevated 30-year risk [25]. Our findings dovetail nicely with these recommendations. If this results in an increase in LLT use in the overall primary prevention population, then based on our data, we would expect a much larger RRR of approximately one-third rather than the average 25 % RRR observed overall in primary prevention populations. It is feasible, therefore, if the present guidelines are better implemented, then in decades to come we may see a significant reduction in incident cardiovascular disease at the population level. Beyond the benefits in the short term, maintaining lower LDL-C levels for longer offers cumulative benefits as recommended in recent guidance [25,70].

Expanding pharmacotherapy to lower-risk populations raises challenges around long-term adherence, which is often suboptimal when perceived short-term benefit is modest, and would require careful risk communication and shared decision-making [71]. In this context, 30-year cardiovascular risk estimates derived from the PREVENT equations may improve targeting by identifying individuals whose short-term risk appears low but who carry a substantially elevated long-term risk profile and stand to derive the greatest cumulative benefit from sustained LDL-C lowering [72]. Better visualization of lifetime benefits, graphically as survival curves or as bar groups, may help to motivate patients instead of the qualitative recommendations, which tend to be the norm. Lipid-lowering therapies, particularly statins, have well-established safety profiles supported by large-scale trials and meta-analyses [21], with serious adverse events uncommon; although modest muscle symptoms and a small excess risk of incident diabetes are observed primarily in those with predisposing risk factors [73]. Non-statin therapies have likewise demonstrated favorable safety and tolerability in clinical trials [74]. Nonetheless, in lower-risk populations where absolute benefit is smaller, long-term adherence and patient preferences become increasingly relevant, underscoring the importance of careful risk communication and shared decision-making when initiating therapy in this group.

Although no lifetime RCTs of LDL-C lowering exist, Mendelian randomization studies have shown that lifelong genetically lower LDL-C concentrations yield much larger benefits (∼50 %) than short-term treatment initiated later in life for each mmol/L drop in LDL-C [5]. However, because Mendelian randomization evidence is observational and reflects lifelong genetic differences from an un-diseased state (birth), RCT evidence remains the most reliable basis for pharmacological recommendations in guidelines [23]. In this context, our study provides trial-level evidence that pharmacological LDL-C lowering initiated in lower-risk populations results in a greater proportional reduction in events, supporting the case of starting simple, low-cost generic monotherapies ideally before the fifth decade, when vascular damage may remain largely modifiable [75].

### 4.1 Strengths and limitations

A key strength of this study was the comprehensive systematic identification of RCTs enrolling populations with low or no ASCVD prevalence. To address heterogeneity in trial design and follow-up, we standardized the 3P-MACE placebo event rate to an annualized rate and all 3P-MACE outcomes to per 1 mmol/L LDL-C reduction, enabling meaningful comparisons across diverse populations. We also compared trial-level findings against the established CTT estimates to enhance the interpretability and generalizability of findings.

However, there are limitations that merit discussion. CTT uses major vascular events and 1 year between group LDL-C difference. By contrast, we used 3P-MACE and a more conservative estimate of LDL-C difference as we averaged the difference at 1 year and at the end of study. LDL-C levels tend to drift up with discontinuation and adherence in both randomized groups in trials; however, where LDL-C differences attenuate due to a differential drop, as in CLEAR OUTCOMES and EWTOPIA 75, this can artificially inflate the treatment difference per 1mmol/L, if medication drop occurred after a censored primary endpoint, making it appear that the treatment effect was larger than average. That said, using 1-year LDL-C differences did not change our findings.

Where trials did not report 3P-MACE, we derived estimates from individual MACE components, and these approximations may differ slightly from direct reporting. As this analysis operates at the trial level, all findings reflect patterns observed across trial populations rather than within individual patients. This approach is appropriate for assessing whether differences in baseline risk between trials are associated with differences in observed treatment effects, but it cannot establish patient-level effect modification. Additionally, using placebo event rate as a trial-level proxy for baseline risk may not accurately reflect individual true baseline risk across heterogenous populations using aggregate data. It should also be noted that the included trials enrolled middle-aged and older adults; thus, our findings relate to LDL-C lowering at lower levels of absolute risk in this age range, rather than to truly early intervention by age. We also excluded one trial that enrolled exclusively renal transplant patients, as cardiovascular events in these patients may be driven by non-atherosclerotic mechanisms [76]. Despite these limitations, the consistency of findings across different scenarios supports their validity, pending prospective confirmation.

Most importantly, even though we published our hypothesis a priori using the PROSPERO framework, prospective validation of our findings in ongoing trials or specifically designing trials based upon our findings are needed. For instance, projections from this review for an ongoing statin trial estimate an LDL-C reduction of 1.31 mmol/L, and predicted relative risk reduction of between 22 % to 48 % for the CTT endpoint based upon the benefits per 1mmol/L for differing placebo event-rates [47]. Ongoing non-statin RCTs include those evaluating an oral PCSK9 inhibitor (enlicitide) and PCSK9 inhibition via small interfering RNA(Inclisiran) could also be used to validate these findings [77,78]. The definition of primary prevention merits discussion briefly. In this study we included VESALIUS-CV patients without known atherosclerosis on imaging [32]. The group of patients with known atherosclerosis (even without prior MI and stroke) [79] behaved more like secondary prevention patients with a lower RRR from evolocumab vs the diabetes no atherosclerosis cohort [32]. This was similar to the greater RRR observed in the primary prevention cohort of CLEAR Outcomes [31].

Our findings have implications for the direction of future research questions, including the need to evaluate whether trial-level patterns of greater proportional benefit at lower baseline risk are confirmed in pooled individual-participant data analyses. If intervention in lower-risk individuals yields compounded long-term benefits in RCTs, in support of Mendelian randomization studies, which already support lifelong low LDL-C [[8], [80], [81]], then long-term trials of one-off interventions, such as PCSK9 gene editing, when started in low-risk cohorts and which reduce LDL-C by approximately 50 %, may be sufficient for many, avoiding the need for combinations of multiple therapies in higher-risk individuals later in life with more atherosclerosis who require 65–85 % LDL-C lowering. Furthermore, outcome trials of 15,000 patients with gene-editing approaches are not viable. However, our observations of greater treatment effects when LDL-C lowering is started in lower-risk populations make it feasible to consider smaller outcome studies with sufficiently long follow-up (10 years) with gene editing approaches beyond simply demonstrating their long-term safety and durability of effect or considering low risk primary prevention trials where large treatment differences from a few hundred events may yield robust findings rather than the several thousands of events required in more advanced disease. Finally, as the field advances, integrating AI-driven risk models, genetic scores, and advanced imaging into clinical decision systems which predict lifetime risk trajectories could help improve our understanding of the magnitude of LDL-C lowering required on a case-by-case basis, moving the field towards truly personalized primary prevention.

## 5 Conclusions

Evidence from this systematic review and meta-analysis demonstrates that LLT initiated in populations with lower risk yields substantially greater relative reductions in cardiovascular events, even with modest LDL-C lowering. By contrast, in high-risk individuals who likely have advanced atherosclerotic cardiovascular disease, greater LDL-C lowering is required to obtain comparable relative benefits. These findings support the evolving evidence base towards initiation of LLT at lower cardiovascular risk as a primary strategy for ASCVD prevention.

## Author agreement

We, the undersigned authors, confirm that this manuscript represents original work that has not been previously published and is not currently under consideration for publication elsewhere. The results of this manuscript have been submitted for consideration for the late-breaker session at the European Atherosclerosis Society Congress in May 2026.

All authors have made substantial contributions to the conception, design, data acquisition, analysis, and interpretation of the work, and have participated in drafting or critically revising the manuscript.

All authors have read and approved the final version of the manuscript submitted for publication. Conflicts of interest and funding sources are declared on the title page.

## Funding

This study received no external funding

## CRediT authorship contribution statement

**Irene Karungi:** Conceptualization, Data curation, Formal analysis, Methodology, Writing – original draft, Writing – review & editing. **Christophe A.T. Stevens:** Conceptualization, Data curation, Formal analysis, Methodology, Writing – review & editing. **Julia Brandts:** Conceptualization, Data curation, Formal analysis, Methodology, Writing – review & editing, Supervision. **Kausik K Ray:** Conceptualization, Data curation, Formal analysis, Methodology, Supervision, Writing – review & editing.

## Declaration of competing interest

IK has participated in a research grant to Imperial from Ultragenyx. CATS has participated in research grants to Imperial from Amgen, Sanofi, Regeneron, Pfizer, Novartis, Merck Sharp & Dohme, Daiichi Sankyo, and Ultragenyx. JB has received research grants from Amgen, AstraZeneca, and Sanofi and speaker honoraria from Amgen, Berlin Chemie, Daiichi Sankyo, Menarini, Novartis, Novo Nordisk, and Sanofi. KKR reports unrestricted research grants (last 3 years) to Imperial College London, Amgen, Sanofi, Regeneron, Daiichi Sankyo, and Ultragenix; Consulting fees from Novartis, Daiichi Sankyo, Kowa, Esperion, Novo Nordisk, MSD, Lilly, Silence Therapeutics, AZ, New Amsterdam Pharma, CLEERLY, EMENDOBIO, SCRIBE, Amarin, Regeneron, Ultragenix. Lecture fees from Novartis, BI, AZ, Novo Nordisk, Viatris, Amarin, Sanofi, Amgen, Esperion, Daiichi Sankyo, USV Pharma, Torrent Pharma, for CME and non-CME symposia at international meetings and stock options from New Amsterdam Pharma, SCRIBE Therapeutic, MONOCYTE Health and PEMI31.

## Appendix Supplementary materials

Image, application 1
