# Supplementary material for: Cardiovascular event rate modifies response to pharmacologic LDL-C lowering in primary prevention: implications of a systematic review and meta-analysis for clinical practice
Source: Am J Prev Cardiol. 2026 May 25;28:101655. doi: 10.1016/j.ajpc.2026.101655 (PMC13330701; doi:10.1016/j.ajpc.2026.101655)
Supplement: Supplementary file 1 [file mmc1.docx]

**SUPPLEMENTARY MATERIAL**

**Cardiovascular event rate modifies response to pharmacologic LDL-C lowering in primary prevention- implications of a systematic review and meta-analysis for clinical practice**

**Authors:**

Irene Karungi, MBChB^a^, Christophe A.T. Stevens, MSc^a^, Julia Brandts, MD^a,b^, Kausik K Ray, MD, FMedSci^a^

| **Index** | | **Page** |
| --- | --- | --- |
| **Supplemental Methods** |  | **2** |
| **Supplemental Table 1:** | Preferred Reporting Items for Systematic Reviews and Meta-Analyses (PRISMA) checklist. | **5** |
| **Supplemental Table 2:** | Eligibility criteria for inclusion and exclusion of primary prevention lipid-lowering trials in this study. | **7** |
| **Supplemental Table 3A:** | Search strategy on OVID EMBASE. | **8** |
| **Supplemental Table 3B:** | Search strategy on OVID MEDLINE. | **9** |
| **Supplemental Table 3C:** | Search strategy on the CENTRAL (Cochrane Library). | **10** |
| **Supplemental Table 4:** | Extracted variables. | **11** |
| **Supplemental Table 5:** | Detailed characteristics of completed trials included in this study. | **12** |
| **Supplemental Table 6:** | Baseline characteristics of completed trials by annual placebo event rate. | **15** |
| **Supplemental Table 7:** | Annual placebo event rates for 5-year baseline risk strata and corresponding HR (99% CI) reported in CTT-2012 and the present analysis. | **16** |
| **Supplemental Table 8:** | Annual placebo event rates and their corresponding standardized HR for CTT-2012 meta-analysis and the present analysis | **16** |
| **Supplemental Table 9A:** | Design, features, and population characteristics of the ongoing trial included in this study. | **17** |
| **Supplemental Table 9B:** | Key predicted outcomes of the ongoing primary prevention trial included in the current analysis. | **17** |
| **Supplemental Figure 1** | PRISMA flow diagram showing selection of eligible trials. | **18** |
| **Supplemental Figure 2** | Standardized RRR of trials not included in CTT-2012 across baseline risk thresholds (event rates) based on CTT-2012 estimates | **20** |
| **Supplemental Figure 3** | RRR of trials not included in CTT-2012 with 0% ASCVD prevalence across baseline risk thresholds (event rates) based on CTT-2012 estimates | **21** |
| **Supplemental Figure 4** | Meta-regression of RRR (y axis) in 3PMACE standardized per 1 mmol/L LDL-C lowering as a function of annualized placebo event rate (x axis) **(S4A)**; absolute LDL-C required to achieve a 25% RRR **(S4B)** across increasing annualized placebo event rate in trials without prevalent established ASCVD. | **22** |
| **Supplemental Figure 5** | Meta-regression of RRR (y axis) in 3PMACE standardized per 1 mmol/L LDL-C lowering as a function of annualized placebo event rate (x axis), among trials reporting between-group change in LDL-C only at 1 year | **24** |
| **Supplemental Figure 6** | Meta-regression of RRR (y axis) in 3PMACE standardized per 1 mmol/L LDL-C lowering as a function of annualized placebo event rate (x axis), stratified by median follow-up duration **(S6A)** and baseline LDL-C **(S6B)**. | **25** |
| **Supplemental Figure 7** | Meta-analysis of HR for 3P-MACE standardized per mmol/L and adjusted to the mean annual placebo event rate (1.7%) by LLT class | **27** |
| **Supplemental Figure 8** | Risk of Bias assessment | **28** |
| **Supplemental Figure 9** | Predicted RRR for CTT endpoint for ongoing trial **(S9A),** and observed RRR for completed trials **(S9B)** by risk threshold (event rates) from CTT-2012 | **29** |
| **Supplemental Figure 10** | Predicted RRR for CTT endpoint for ongoing trial and observed RRR for completed trials by each risk category (event rate) from CTT-2012 | **31** |
| **References** |  | **33** |

**Supplemental Methods**

**Data sources and search strategy**

A comprehensive systematic search was conducted in Embase (Ovid), Medline (Ovid), and the Cochrane Central Register of Controlled Trials (CENTRAL) via the Cochrane Library. Structured search strategies were developed and conducted for each database from inception to November 3, 2025, as detailed in **Supplemental Tables 3a,** **3b, and 3c**. The searches were restricted to studies involving humans. Additionally, backward citation searching (reference list checking) of included studies was performed to identify any additional eligible studies.

**Data extraction**

Two reviewers (CATS and IK) independently screened titles and abstracts of all identified records using Covidence systematic review software. Full-text articles of potentially eligible studies were retrieved and independently assessed by three reviewers (JB, CATS, IK), and any discrepancies were resolved through discussion and consensus with an additional reviewer (KKR, JB, CATS, IK). Data from the final set of eligible trials were extracted independently by two reviewers (CATS and IK) using a standardized extraction form. Extracted variables are provided in **Supplemental Table 4**.

**Baseline characteristics**

Baseline characteristics extracted from each trial included age, sex, body mass index (BMI), systolic blood pressure (SBP), diastolic blood pressure (DBP), baseline LDL-C, glomerular filtration rate (GFR), prevalence of hypertension, diabetes mellitus, chronic kidney disease, smoking, and prior ASCVD. To summarise each trial’s study population, control, and intervention arms were pooled. Continuous variables reported as means (SD) or medians (IQR) were pooled across study arms using sample-size-weighted pooled means (SD) and pooled medians (IQR). Categorical variables were combined by summing event counts across both arms and expressed as proportions. An overall weighted summary was calculated across all trials. To obtain this for continuous variables, medians (IQR) were first converted to estimated means (SD) using the method described by Wan et al.^1^, then pooled across trials using sample-size weighting. Categorical variables were expressed as overall proportions. Additionally, baseline characteristics were stratified by overall trial annual placebo event rate categories (<1%, 1-2%, and ≥2%), with differences across strata assessed using weighted ANOVA for continuous variables and chi-square tests for categorical variables.

**Study outcomes**

The primary outcome for this study was 3P-MACE, defined as the composite of non-fatal myocardial infarction (MI), non-fatal stroke, and cardiovascular death. For trials that did not directly report 3P-MACE, the composite was estimated using inverse-variance weighting of the individual 3P-MACE components. Additionally, in trials (AFCAPS/TeXCAPS, ASCOT-LLA, and ASPEN) where MI and stroke counts were combined as fatal/nonfatal, the 3P-MACE and annual placebo event rate were calculated using the combined counts, excluding cardiovascular death, as most of these events would be captured in the nonfatal counts. Additionally, the composite endpoint for PROSPER comprised non-fatal MI, fatal and nonfatal stroke, and CHD death, and this endpoint was retained as reported.

**Subgroup and sensitivity analyses**

Linear regression was used to generate best-fit regression lines for the CTT-2012 relative risk reduction (RRR) per 1 mmol/L LDL-C lowering across annual placebo event rates for participants without established atherosclerotic cardiovascular disease (ASCVD)^2^. Where CTT risk strata had overlapping estimates, inverse variance weighting was used to pool and derive a single hazard ratio. Trial-level estimates for RCTs (published after CTT-2012 or not reported in CTT-2012) were plotted against the linear regressions derived from CTT-2012 estimates. For these trials, where the CTT endpoint was available, it was used; otherwise, 3P-MACE were used for plotting. Subgroup analyses included using standardized 3P-MACE, restricting to trials with 0% prevalence of clinically evident ASCVD, and splitting by individual CTT-2012 annual placebo event rates. Mixed-effects meta-regression analysis in R (V4.3.2) was conducted on all available trial-level data to assess effect modification of the annual placebo event rate on the standardized 3P-MACE RRR. Sensitivity analyses were restricted to trials with 0% prevalence of clinically evident ASCVD and for trials reporting absolute LDL-C change between groups at 1 year. Using trial-level estimates, we also estimated the LDL-C reduction required to achieve a 25% RRR in 3P-MACE across risk strata with sensitivity analyses also restricted to trials with 0% prevalence of clinically evident ASCVD.

**Predictive modeling for the ongoing trial**

For the ongoing trial, predictions for RRR were calculated using the equation: 1-HR ∆LDL-C. Where HR represents the hazard ratio per 1 mmol/L LDL-C reduction reported in the CTT-2012 for the corresponding baseline risk category^2^, and ∆LDL-C represents the trial’s mean LDL-C reduction, estimated by applying the percentage LDL-C reduction reported for the same drug and dose in previous clinical trials or meta-analyses to the trial’s baseline LDL-C. The equation was adapted from a health-system-level intervention simulation study^3^.

**Supplemental Table 1: Preferred Reporting Items for Systematic Reviews and Meta-Analyses (PRISMA) checklist.**

| **Section and Topic** | **Item #** | **Checklist item** | **Location where item is reported** |
| --- | --- | --- | --- |
| **TITLE** | | |  |
| Title | 1 | Identify the report as a systematic review. | Pg. 1 |
| **ABSTRACT** | | |  |
| Abstract | 2 | See the PRISMA 2020 for Abstracts checklist. | Pg. 2 |
| **INTRODUCTION** | | |  |
| Rationale | 3 | Describe the rationale for the review in the context of existing knowledge. | Pg. 5,6 |
| Objectives | 4 | Provide an explicit statement of the objective(s) or question(s) the review addresses. | Pg. 5,6 |
| **METHODS** | | |  |
| Eligibility criteria | 5 | Specify the inclusion and exclusion criteria for the review and how studies were grouped for the syntheses. | Pg. 6,7, and Supplemental Table 2 |
| Information sources | 6 | Specify all databases, registers, websites, organisations, reference lists and other sources searched or consulted to identify studies. Specify the date when each source was last searched or consulted. | Pg. 7 |
| Search strategy | 7 | Present the full search strategies for all databases, registers and websites, including any filters and limits used. | Pg. 7, Supplemental Table 2 |
| Selection process | 8 | Specify the methods used to decide whether a study met the inclusion criteria of the review, including how many reviewers screened each record and each report retrieved, whether they worked independently, and if applicable, details of automation tools used in the process. | Pg. 7,8, and Supplemental Tables 3a, 3b, and 3c |
| Data collection process | 9 | Specify the methods used to collect data from reports, including how many reviewers collected data from each report, whether they worked independently, any processes for obtaining or confirming data from study investigators, and if applicable, details of automation tools used in the process. | Pg. 7 |
| Data items | 10a | List and define all outcomes for which data were sought. Specify whether all results that were compatible with each outcome domain in each study were sought (e.g. for all measures, time points, analyses), and if not, the methods used to decide which results to collect. | Pg. 7,8, Supplemental Pg. 2,3 and Supplemental Table 4 |
|  | 10b | List and define all other variables for which data were sought (e.g. participant and intervention characteristics, funding sources). Describe any assumptions made about any missing or unclear information. | Pg. 7,8, Supplementary Pg. 2,3 and Supplemental Table 4 |
| Study risk of bias assessment | 11 | Specify the methods used to assess risk of bias in the included studies, including details of the tool(s) used, how many reviewers assessed each study and whether they worked independently, and if applicable, details of automation tools used in the process. | Pg. 7 |
| Effect measures | 12 | Specify for each outcome the effect measure(s) (e.g. risk ratio, mean difference) used in the synthesis or presentation of results. | Pg. 8,9 Supplementary Pg. 2,3 |
| Synthesis methods | 13a | Describe the processes used to decide which studies were eligible for each synthesis (e.g. tabulating the study intervention characteristics and comparing against the planned groups for each synthesis (item #5)). | Pg. 8,9, Supplementary Pg. 2,3,4 |
|  | 13b | Describe any methods required to prepare the data for presentation or synthesis, such as handling of missing summary statistics, or data conversions. | Pg. 8,9, Supplementary Pg. 2,3,4 |
|  | 13c | Describe any methods used to tabulate or visually display results of individual studies and syntheses. | Pg. 8,9, Supplementary Pg. 2,3,4 |
|  | 13d | Describe any methods used to synthesize results and provide a rationale for the choice(s). If meta-analysis was performed, describe the model(s), method(s) to identify the presence and extent of statistical heterogeneity, and software package(s) used. | Supplementary Pg. 4 |
|  | 13e | Describe any methods used to explore possible causes of heterogeneity among study results (e.g. subgroup analysis, meta-regression). | Pg. 9, Supplementary Pg.3 |
|  | 13f | Describe any sensitivity analyses conducted to assess robustness of the synthesized results. | Supplementary Pg. 3,4 |
| Reporting bias assessment | 14 | Describe any methods used to assess risk of bias due to missing results in a synthesis (arising from reporting biases). | NA |
| Certainty assessment | 15 | Describe any methods used to assess certainty (or confidence) in the body of evidence for an outcome. | NA |
| **RESULTS** | | |  |
| Study selection | 16a | Describe the results of the search and selection process, from the number of records identified in the search to the number of studies included in the review, ideally using a flow diagram. | Pg. 10, Supplemental Figure 1, |
|  | 16b | Cite studies that might appear to meet the inclusion criteria, but which were excluded, and explain why they were excluded. | Pg. 19 |
| Study characteristics | 17 | Cite each included study and present its characteristics. | Pg.10, Table 1 |
| Risk of bias in studies | 18 | Present assessments of risk of bias for each included study. | Supplemental Figure 8 |
| Results of individual studies | 19 | For all outcomes, present, for each study: (a) summary statistics for each group (where appropriate) and (b) an effect estimate and its precision (e.g. confidence/credible interval), ideally using structured tables or plots. | Pg. 10,11, Table 2 |
| Results of syntheses | 20a | For each synthesis, briefly summarise the characteristics and risk of bias among contributing studies. | Pg. 13 |
|  | 20b | Present results of all statistical syntheses conducted. If meta-analysis was done, present for each the summary estimate and its precision (e.g. confidence/credible interval) and measures of statistical heterogeneity. If comparing groups, describe the direction of the effect. | Pg. 11,12 Table 2, Figures 1A,1B,2A,2B, supplemental figures 2,3,4,5A,5B,6A,6B,7,8,10,11 |
|  | 20c | Present results of all investigations of possible causes of heterogeneity among study results. | NA |
|  | 20d | Present results of all sensitivity analyses conducted to assess the robustness of the synthesized results. | supplemental figures 2,3,4,5, 7, |
| Reporting biases | 21 | Present assessments of risk of bias due to missing results (arising from reporting biases) for each synthesis assessed. | NA |
| Certainty of evidence | 22 | Present assessments of certainty (or confidence) in the body of evidence for each outcome assessed. | NA |
| **DISCUSSION** | | |  |
| Discussion | 23a | Provide a general interpretation of the results in the context of other evidence. | Pg. 14 |
|  | 23b | Discuss any limitations of the evidence included in the review. | Pg. 18 |
|  | 23c | Discuss any limitations of the review processes used. | Pg. 18 |
|  | 23d | Discuss implications of the results for practice, policy, and future research. | Pg. 18 |
| **OTHER INFORMATION** | | |  |
| Registration and protocol | 24a | Provide registration information for the review, including register name and registration number, or state that the review was not registered. | [CRD420251155320](https://www.crd.york.ac.uk/PROSPERO/view/CRD420251155320) |
|  | 24b | Indicate where the review protocol can be accessed, or state that a protocol was not prepared. | Protocol available on PROSPERO |
|  | 24c | Describe and explain any amendments to information provided at registration or in the protocol. | Additional methodological and analytical refinements to improve consistency and transparency. |
| Support | 25 | Describe sources of financial or non-financial support for the review, and the role of the funders or sponsors in the review. | N/A |
| Competing interests | 26 | Declare any competing interests of review authors. | 21 |
| Availability of data, code and other materials | 27 | Report which of the following are publicly available and where they can be found: template data collection forms; data extracted from included studies; data used for all analyses; analytic code; any other materials used in the review. | N/A |

**Supplemental Table 2: Eligibility criteria for inclusion and exclusion of primary prevention lipid-lowering trials in this study**

| Domain | Inclusion Criteria | Exclusion Criteria |
| --- | --- | --- |
| Population | 1. Primary prevention populations comprising participants without established or clinically manifest atherosclerotic cardiovascular disease (ASCVD), defined as a history of coronary artery disease (including myocardial infarction or coronary revascularization), cerebral vascular disease (including stroke or transient ischaemic attack), or other atherosclerotic complications).  2. Mixed populations comprising participants without (primary prevention) or with (secondary prevention) established or clinically manifest ASCVD. These will be eligible only if either:   - Primary prevention results were reported separately in the main trial or in a post-hoc analysis, or - Participants with prior ASCVD comprised ≤20% of the study population. | 1. Populations restricted to secondary prevention.  2. Mixed populations where primary prevention results could not be distinguished, or more than 20% of participants had prior ASCVD at baseline. |
| Intervention | 1. Statin-based lipid-lowering therapy  2. Non-statin-based lipid-lowering therapy  3. Combination consisting of statin or non-statin-based lipid-lowering therapy. | 1. Interventions consisting of fibrates or omega-3 fatty acids.  2. Non-pharmacological interventions, for example, lifestyle modifications. |
| Comparator | 1. Comparator consisting of placebo or usual care. | 2. comparator consisting of alternative lipid-lowering regimens. |
| Outcomes | 1. RCTs reporting 3P-MACE (non-fatal MI, non-fatal stroke, cardiovascular death) or  2. RCTs reporting the individual MACE component outcomes enabling calculation of 3P-MACE. | 1. RCTs with inadequate reporting of 3P-MACE.  2. Post-hoc analyses reporting 3P-MACE, however, are from primary prevention trials, which were included in CTT-2012.  3. Post-hoc analyses from primary prevention trials that report non-MACE endpoints. |
| Study design | 1. Completed RCTs with published results.  2. Ongoing RCTs with published baseline data.  3. RCTs with ≥1,000 enrolled participants  4. RCTs with more than 1 year of median follow-up |  |

ASCVD; atherosclerotic cardiovascular disease, CTT-2012; Cholesterol Treatment Trialists’ Collaboration, 2012, MACE; major adverse cardiovascular disease, MI; myocardial infarction, RCT; randomized controlled trial.

**Supplemental Table 3A: Search strategy on OVID EMBASE**

| Disease terms | exp Cardiovascular Disease/  OR cardiovascular outcome*.mp. OR cardiovascular event*.mp. OR cardiovascular death*.mp. OR myocardial infarction*.mp. OR stroke*.mp. OR transient isch?emic attack*.mp. OR coronary revasculari?ation*.mp. OR PCI*.mp. OR CABG*.mp. OR ASCVD*.mp. OR atherosclerotic CVD*.mp. OR atherosclerotic cardiovascular disease*.mp. OR major cardiovascular event*.mp. OR major adverse cardiovascular event*.mp. |
| --- | --- |
| AND | |
| Population terms | exp Primary Prevention/  OR cardiovascular prevention*.mp. OR (primary adj3 prevent*).mp. OR (asymptomatic adj3 cardiovas*).mp. OR (without prior adj3 myocardial infarction*).mp. OR (without prior adj3 stroke*).mp. OR (without prior adj3 cardiovas*).mp. OR (no history adj3 cardiovas*).mp. OR (no history adj3 myocardial infarction*).mp. OR (no history adj3 stroke*).mp. OR (no history adj3 coronary revasculari?ation*).mp OR (low adj3 cardiovas*).mp. OR (intermediate adj3 cardiovas*).mp. |
| AND | |
| Intervention terms | exp antilipemic agent/  OR lipid lowering*.mp. OR cholesterol lowering*.mp. OR hypolipidemic agent*.mp. OR  exp hydroxymethylglutaryl-coenzyme A reductase inhibitor/ OR statin*.mp. OR atorvastatin*.mp. OR rosuvastatin*.mp. OR simvastatin*.mp. OR pravastatin*.mp. OR pitavastatin*.mp. OR fluvastatin*.mp. OR lovastatin*.mp. OR  exp PCSK9 inhibitor/ OR evolocumab*.mp. OR alirocumab*.mp. OR  exp ezetimibe/ OR cholesterol absorption inhibitor*.mp. OR ezetimibe plus statin*.mp. OR  ATP citrate lyase inhibitor*.mp. OR ACL inhibitor*.mp. OR bempedoic acid*.mp. OR inclisiran*.mp. OR bempedoic acid plus statin*.mp. OR non statin*.mp. OR statin intolerant*.mp. OR ANGPTL3 inhibitor*.mp. OR evinacumab*.mp. OR bile acid sequestrant*.mp. OR cholestyramine*.mp. OR colestipol*.mp. OR colesevelam*.mp. OR CETP inhibitor*.mp. OR obicetrapib*.mp. OR anacetrapib*.mp. OR probucol*.mp. |
| AND | |
| Study design terms | exp randomized controlled trial/  OR randomized clinical trial OR controlled clinical trial.pt. OR double blind*.mp. OR placebo controlled*.mp. OR RCT*.mp. |
| NOT | |
| Non-human (human studies filter) | exp animals/ not human. |

##

**Supplemental Table 3B: Search strategy on OVID MEDLINE**

| Disease terms | exp Cardiovascular Diseases/  OR cardiovascular outcome*.mp. OR cardiovascular event*.mp. OR cardiovascular death*.mp. OR myocardial infarction*.mp. OR stroke*.mp. OR transient isch?emic attack*.mp. OR coronary revasculari?ation*.mp. OR PCI*.mp. OR CABG*.mp. OR ASCVD*.mp. OR atherosclerotic CVD*.mp. OR atherosclerotic cardiovascular disease*.mp. OR major cardiovascular event*.mp. OR major adverse cardiovascular event*.mp. |
| --- | --- |
| AND | |
| Population terms | exp Primary Prevention/  OR cardiovascular prevention*.mp. OR (primary adj3 prevent*).mp. OR (asymptomatic adj3 cardiovas*).mp. OR (without prior adj3 myocardial infarction*).mp. OR (without prior adj3 stroke*).mp. OR (without prior adj3 cardiovas*).mp. OR (no history adj3 cardiovas*).mp. OR (no history adj3 myocardial infarction*).mp. OR (no history adj3 stroke*).mp. OR (no history adj3 coronary revasculari?ation*).mp OR (low adj3 cardiovas*).mp. OR (intermediate adj3 cardiovas*).mp. |
| AND | |
| Intervention terms | exp Hypolipidemic Agents/  OR lipid lowering*.mp. OR cholesterol lowering*.mp. OR antilipemic agent*.mp. OR  exp Hydroxymethylglutaryl-CoA Reductase Inhibitors/ OR statin*.mp. OR atorvastatin*.mp. OR rosuvastatin*.mp OR simvastatin*.mp. OR pravastatin*.mp. OR pitavastatin*.mp. OR fluvastatin*.mp. OR lovastatin*.mp. OR  exp PCSK9 Inhibitors/ OR evolocumab*.mp. OR alirocumab*.mp. OR  exp Ezetimibe/ OR cholesterol absorption inhibitor*.mp. OR ezetimibe plus statin*.mp. OR  ATP citrate lyase inhibitor*.mp. OR ACL inhibitor*.mp. OR bempedoic acid*.mp. OR inclisiran*.mp. OR bempedoic acid plus statin*.mp. OR non statin*.mp. OR statin intolerant*.mp. OR ANGPTL3 inhibitor*.mp. OR evinacumab*.mp. OR bile acid sequestrant*.mp. OR cholestyramine*.mp. OR colestipol*.mp. OR colesevelam*.mp. OR CETP inhibitor*.mp. OR obicetrapib*.mp. OR anacetrapib*.mp. OR probucol*.mp. |
| AND | |
| Study design terms | Randomized Controlled Trial/  OR randomized controlled trial.pt. OR controlled clinical trial.pt. OR double blind*.mp. OR placebo controlled*.mp. OR RCT*.mp. |
| NOT | |
| Non-human (human studies filter) | exp animals/ not human |

##

**Supplemental Table 3C: Search strategy on the CENTRAL (Cochrane Library)**

| Disease terms | MeSH descriptor: [Cardiovascular Diseases] explode all trees  OR "cardiovascular outcome*" OR "cardiovascular event*" OR "cardiovascular death*" OR "myocardial infarction*" OR stroke* OR "transient isch?emic attack*" OR "coronary revasculari?ation*" OR PCI* OR CABG* OR ASCVD* OR "atherosclerotic CVD*" OR "atherosclerotic cardiovascular disease*" OR "major cardiovascular event*" OR "major adverse cardiovascular event*" |
| --- | --- |
| AND | |
| Population terms | MeSH descriptor: [Primary Prevention] explode all trees  OR "cardiovascular prevention*" OR (primary NEAR/3 prevent*) OR (asymptomatic NEAR/3 cardiovas*) OR (“without prior” NEAR/3 “myocardial infarction*”) (“without prior” NEAR/3 stroke*) (“without prior” NEAR/3 cardiovas*) OR (“no history” NEAR/3 “myocardial infarction*”) OR (“no history” NEAR/3 stroke*) OR (“no history” NEAR/3 “coronary revasculari?ation*”) OR (low NEAR/3 cardiovas*) OR (intermediate NEAR/3 cardiovas* |
| AND | |
| Intervention terms | MeSH descriptor: [Hypolipidemic Agents] explode all trees  OR "lipid lowering*" OR "cholesterol lowering*" OR "antilipemic agent*" OR  MeSH descriptor: [Statins, HMG CoA] explode all trees  OR statin* OR atorvastatin* OR rosuvastatin* OR simvastatin* OR pravastatin* OR pitavastatin* OR fluvastatin* OR lovastatin* OR  MeSH descriptor: [PCSK9 Inhibitors] explode all trees OR evolocumab* OR alirocumab* OR  MeSH descriptor: [Ezetimibe] explode all trees OR "cholesterol absorption inhibitor*" OR "ezetimibe plus statin*" OR "ATP citrate lyase inhibitor*" OR "ACL inhibitor*" OR "bempedoic acid*" OR inclisiran* OR "bempedoic acid plus statin*" OR "non statin*" OR "statin intolerant*" OR "ANGPTL3 inhibitor*" OR evinacumab* OR "bile acid sequestrant*" OR cholestyramine* OR colestipol* OR colesevelam* OR "CETP inhibitor*" OR obicetrapib* OR anacetrapib* OR probucol |
| AND | |
| Study design terms | MeSH descriptor: [Randomized Controlled Trial] explode all trees  OR “randomized controlled trial*” OR “controlled clinical trial*” OR “double blind*” OR “placebo controlled*” OR RCT* |

##

**Supplemental Table 4: Extracted variables**

| **Study characteristics** |
| --- |
| Authors |
| Year published |
| Trial name |
| Trial site |
| Trial design |
| Number of study arms |
| Sample size |
| Name and dose of lipid-lowering therapy |
| Type of control group used |
| Mean (SD) duration of follow-up |
| Median (IQR) duration of follow-up |
| Criteria for determining cardiovascular baseline risk of the trial population |
| **Participant characteristics** |
| Number (%) of male participants |
| Number (%) of female participants |
| Mean (SD) age in years |
| Median (IQR) age in years |
| Mean (SD) BMI in Kg/m^2^ |
| Median (IQR) BMI in Kg/m^2^ |
| Mean (SD) SBP and DBP (mmHg) |
| Median (IQR) SBP and DBP (mmHg) |
| Number (%) of participants with hypertension |
| Number (%) of participants with diabetes |
| Number (%) of participants with CKD |
| Mean (SD) GFR in mL/min/1.73m² |
| Median (IQR) GFR in mL/min/1.73m² |
| Number (%) of participants who are smokers |
| Number (%) of participants with ASCVD |
| LDL-C at baseline |
| LDL-C at 1 year |
| LDL-C at study completion |
| LDL-C percentage reduction at 1 year and at study completion |
| Event rate for 3P MACE in the placebo group |
| **Outcomes** |
| Event count, HR/RRR for 3P-MACE |
| Event count, HR/RRR for components of 3P-MACE (non-fatal myocardial infarction, non-fatal stroke, cardiovascular death) |

3P-MACE; 3 point major adverse cardiovascular disease, ASCVD; atherosclerotic cardiovascular disease, BMI; body mass index, CKD; chronic kidney disease, DBP; diastolic blood pressure, DM; diabetes mellitus, IQR; interquartile range, GFR; glomerular filtration rate, LDL-C; low density lipoprotein cholesterol, , RRR; relative risk reduction, SBP; systolic blood pressure, SD; standard deviation.

##

**Supplemental Table 5: Detailed characteristics of completed statin and non-statin primary prevention trials included in the current analysis**

| **Trial** | **Intervention** | **Background LLT n (%)** | **Trial site(s)** | **Criteria for risk assessment or participant enrollment** |
| --- | --- | --- | --- | --- |
| WOSCOPS | Placebo | 0 | United Kingdom | Men 45-64 years, recruited via population screening with LDL-C levels 4.5mmol/l, without prior MI. |
|  | Pravastatin (40mg) | 0 |  |  |
| AFCAPS/  TeXCAPS | Placebo | 0 | United states | Men and women with TC 180 to 264 mg/dl, LDL cholesterol 130 to 190 mg/dl, HDL cholesterol ≤45 mg/dl for men, and ≤47 mg/dl for women, and triglycerides ≤400 mg/dl. Excluded individuals with a prior history of MI, angina, claudication, cerebrovascular accident, or TIA. Also excluded those aged >73 or <45 (men) or <55 (women) years or with secondary hyperlipoproteinemia, nephrotic syndrome, uncontrolled or insulin-dependent DM, or uncontrolled hypertension. |
|  | Lovastatin (20-40mg) | 0 |  |  |
| PROSPER | Placebo | 0 | United Kingdom (Scotland), Ireland, Netherlands | The primary prevention group included adults without a prior history of pre-existing vascular disease (coronary, cerebral, or peripheral) but with a raised risk of disease due to smoking, hypertension, or diabetes. Their plasma total cholesterol needed to be 4·0–9·0 mmol/L and their triglyceride concentrations less than 6·0 mmol/L. |
|  | Pravastatin (40mg) | 0 |  |  |
| ASCOT-LLA | Placebo | 51  (1.0) | United Kingdom, Ireland, Denmark, Finland, Iceland, Norway, Sweden | Individuals aged 40 -79 years with either untreated hypertension, defined as SBP of 160 mm Hg or more, DBP of 100 mm Hg or more, or both, or treated hypertension with SBP of 140 mm Hg or more, DBP of 90 mm Hg or more, or both. Patients included if with TC ≤6·5 mmol/L, not on LLT and with ≥ CVD risk factors: LVH, other specified ECG abnormalities, type 2 diabetes, PAD, previous stroke or TIA, male sex, age 55 years or older, microalbuminuria or proteinuria, smoking, TC: HDL ratio≥ 6, or premature family history of CHD. Exclusions included previous MI, treated angina, cerebrovascular event, fasting triglycerides >4·5 mmol/L, HF, and uncontrolled arrhythmias. |
|  | Atorvastatin (10mg) | 41  (0.8) |  |  |
| CARDS | Placebo | 0 | United Kingdom, Ireland | Men and women aged 40–75 years with type 2 diabetes mellitus with at least one or more of the following: a history of hypertension, defined as receiving antihypertensive treatment or having systolic blood pressure of 140 mm Hg or greater or diastolic blood pressure of 90 mm Hg or greater on at least two successive occasions; retinopathy, microalbuminuria or macroalbuminuria, or currently smoking. Patients were ineligible if they had history of MI, angina, coronary vascular surgery, cerebrovascular accident, or severe peripheral vascular disease. Mean serum LDL-cholesterol concentration during baseline visits had to be 4·14 mmol/L or lower and serum triglycerides 6·78 mmol/L or less. |
|  | Atorvastatin (10mg) | 0 |  |  |
| MEGA | NCEP step 1 diet | 0 | Japan | Enrolled men and postmenopausal women aged 40–70 years with a bodyweight of 40 kg or more and hypercholesterolaemia (TC 5·69–6·98 mmol/L). Major exclusion criteria were familial hypercholesterolaemia and a history of coronary heart disease or stroke. |
|  | NCEP step 1 diet + Pravastatin (10-20mg) | 0 |  |  |
| ASPEN | Placebo | 0 | Australia, Austria, Canada, Finland, France, Germany, Italy, the Netherlands, New Zealand, Norway, South Africa, Spain, Switzerland, and the U.S | Male and female subjects, aged 40–75 years, with type 2 diabetes ≥3 years before screening. LDL cholesterol criteria were *1*) LDL cholesterol ≤140 mg/dl if subjects had documented myocardial infarction or an interventional procedure >3 months before screening, or *2*) LDL cholesterol ≤4.1 mmol/l if not, Triglycerides ≤600 mg/dl at all visits. Exclusions were type 1 diabetes, MI, interventional procedure, or episodes of unstable angina ≤3 months before screening; HbA_1c_ (A1C) >10%; active liver disease or hepatic dysfunction, severe renal dysfunction or nephrotic syndrome; congestive heart failure treated with digoxin; creatine phosphokinase ≥3 × the upper limit of normal; blood pressure >160/100 mmHg; BMI >35 kg/m^2^; abuse of alcohol and/or drugs. |
|  | Atorvastatin (10mg) | 0 |  |  |
| JUPITER | Placebo | 0 | Argentina, Belgium, Bulgaria, Canada, Chile, Colombia, Costa Rica, Denmark, El Salvador, Estonia, Germany, Israel, Netherlands, Norway, Panama, Poland, Romania, Russia, South Africa, Switzerland, United Kingdom, Uruguay, United States, Venezuela | Men aged ≥ 50 years and women aged ≥ 60 years without prior ASCVD were eligible if at screening, they had an LDL-C <3.4 mmol/L, HS-CRP ≥ 2.0 mg/L, and triglycerides <5.6 mmol/L. Exclusions included previous or current LLT use, use of postmenopausal hormone-replacement therapy, hepatic dysfunction (ALT 2x ULN), CK>3x ULN, creatinine> 2.0mg/dl, diabetes, uncontrolled hypertension (SBP>190 mm Hg or DBP >100 mm Hg), cancer within 5 years before enrollment (with the exception of basal-cell or squamous-cell carcinoma of the skin), uncontrolled hypothyroidism (TSH >1.5 ULN), and a recent history of alcohol or drug abuse |
|  | Rosuvastatin (20mg) | 0 |  |  |
| SEAS | Placebo | 0 | Norway, Sweden, Denmark, Finland, Germany, the United Kingdom, and Ireland | Trial enrolled patients with asymptomatic aortic stenosis, defined as aortic valve thickening using echocardiographic evaluation accompanied by a Doppler-measured aortic peak flow velocity of≥2.5 and ≤4.0 m/s, representing mild to moderate aortic stenosis. LDL-Cholesterol <6 mmol/L (232 mg/dl). |
|  | Simvastatin (40mg) and ezetimibe (10mg) | 0 |  |  |
| SHARP | Placebo | 0 | Australia, Austria, Canada, China, Czech Republic, Denmark, Finland, France, Germany, Malaysia, Netherlands, New Zealand, Norway, Poland, Sweden, Thailand, United Kingdom | Men or women aged ≥40 years with a history of CKD defined as; predialysis (creatinine ≥1.7 mg/dL in men or ≥1.5 mg/dL in women) or Dialysis (hemodialysis or peritoneal dialysis). Excluded individuals with a definite history of MI or a coronary revascularization procedure, functioning renal transplant or living donor renal transplant planned, a less than 2 months since presentation as an acute uremic emergency, a definite history of chronic liver disease or abnormal liver function (ie, ALT >1.5× ULN or, if ALT not available, AST >1.5× ULN) (patients with a history of hepatitis were eligible if these limits are not exceeded), an evidence of active inflammatory muscle disease (eg, dermatomyositis, polymyositis) |
|  | Simvastatin (20mg) and ezetimibe (10mg) | 0 |  |  |
| HOPE-3 | Placebo | 6  (0.1) | Argentina, Australia, Brazil, Canada, China, Colombia, Ecuador, Czech Republic, Hungary, India, Israel, Malaysia, Netherlands, Philippines, Russia, Slovakia, South Africa, Sweden, United Kingdom, Ukraine | Trial enrolled individuals with an annual risk of major cardiovascular events of approximately 1% (men older than 55 years and women older than 65 years with at least 1 additional CV risk factor, a moderately elevated waist-to-hip ratio, a history of low HDL-C, recent tobacco use, dyslipidemia, a family history of premature cardiovascular disease, or early renal dysfunction). Women aged 60 or older who had at least 2 such risk factors were also included. The INTERHEART score was used to determine participant cardiovascular risk. |
|  | Rosuvastatin (10mg) | 11  (0.2) |  |  |
| ALLHAT-LLT | NCEP step 1 diet | 0 | United states | The trial enrolled ambulatory adults 55 years and older having stage 1 or 2 hypertension with at least one additional CHD risk factor. For individuals without known CHD, a fasting LDL-C level of 120 to 189 mg/dL and a fasting triglyceride level lower than 350 mg/dL were required. |
|  | NCEP step 1 diet and Pravastatin (40mg) | 0 |  |  |
| TRACE-RA | Placebo | 0 |  | Patients were eligible if they fulfilled the American College of Rheumatology 1987 criteria for RA, were >50 years of age, or had an RA disease duration of >10 years. Excluded patients on LLT, with DM or contraindications to statins (myopathy). |
|  | Atorvastatin (40mg) | 0 |  |  |
| EWTOPIA 75 | Dietary counseling | 0 | Japan | Individuals aged ≥75 years with serum LDL-C level ≥140 mg/dL, and at least 1 risk factor such as DM, hypertension, low HDL-C, hypertriglyceridemia, current smoker, a history of symptomatic, imaging-confirmed stroke, or peripheral artery disease were enrolled. No formal risk calculator was used to assess background risk. |
|  | Dietary counseling and ezetimibe (10mg) | 0 |  |  |
| REPRIEVE | Placebo | 0 | US, Canada, Spain, Brazil, Peru, Haiti, Thailand, India, South Africa, Uganda, Botswana, Zimbabwe | 40-75-year-old individuals with HIV infection, stable on antiretroviral therapy. All with a low-to-moderate risk of atherosclerotic cardiovascular disease, as determined by the score on the American Heart Association and American College of Cardiology 2013 Pooled Cohort Equation risk calculator a risk of up to 15% for LDL-C 1.81 mmol/L. Excluded those on LLT or known ASCVD. |
|  | Pitavastatin (4mg) | 0 |  |  |
| CLEAR Outcomes | Placebo | 568  (27) | Argentina, Australia, Austria, Belgium, Brazil, Bulgaria, Canada, Chile, Colombia, Croatia, Czech Republic, Denmark, Estonia, Germany, Hungary, India, Latvia, Lithuania, Mexico, Netherlands, New Zealand, Poland, Romania, Russia, Serbia, Slovakia, South Africa, Spain, Turkey, Ukraine, the United Kingdom, and the US. | The trial enrolled individuals who were 18 to 85 years with an LDL-C ≥ 2.59 mmol/L with high risk of CVD based on Reynolds Risk Score or E-SCORE, or a coronary artery calcium score greater than 400 Agatston units, or the presence of either type 1 or 2 diabetes in women older than 65 years or men older than 60 years. |
|  | Bempedoic acid (180mg) | 578  (28) |  |  |
| VESALIUS-CV | Placebo | 5609 (92) | Argentina, Australia, Austria, Belgium, Brazil, Bulgaria, Canada, China, Czech Republic, Denmark, Estonia, Finland, France, Germany, Greece, Hungary, Iceland, Italy, Latvia, Lithuania, Mexico, Netherlands, Poland, Portugal, Romania, Russian Federation, Slovakia, South Korea, Spain, Sweden, Taiwan, Turkey, United Kingdom, Ukraine, US. | Eligibility required age 50 (men) or 55 (women) to 79 years old with an LDL-C ≥2.3 mmol/L, non-HDL-C ≥3.1 mmol/L, or  ApoB ≥1.56 µmol/L on at least 2 weeks of stable, optimized LLT and at high risk of CVD based on age, use of LLT, lipids parameters, and High-risk diabetes with microvascular disease (UACR ≥30 mg/g or eGFR <60), ≥10-year duration, insulin use, or high-risk criteria without known significant atherosclerosis, defined as no prior arterial revascularization, no known arterial stenosis 50% or greater, and no known coronary artery calcium score 100 Agatston units or greater. |
|  | Evolocumab (140mg) | 5641 (92) |  |  |

ABI; ankle–brachial index, HbA1c; glycated haemoglobin, ALLHAT-LLT; antihypertensive and lipid-lowering treatment to prevent heart attack trial–lipid-lowering trial, ALT; alanine aminotransferase, ApoB; apolipoprotein B, ASCVD; atherosclerotic cardiovascular disease, AST; aspartate aminotransferase, BMI; body mass index, CAD; coronary artery disease, CHD; coronary heart disease, CK; creatine kinase, CKD; chronic kidney disease, CLEAR Outcomes; cholesterol lowering via bempedoic acid, an ACL-inhibiting regimen, CV; cardiovascular, CVD; cardiovascular disease, DBP; diastolic blood pressure, DM; diabetes mellitus, ECG; electrocardiogram, eGFR; estimated glomerular filtration rate, EWTOPIA 75; ezetimibe lipid-lowering trial on prevention of atherosclerotic cardiovascular disease in 75 or older, FH; familial hypercholesterolaemia, HDL-C; high-density lipoprotein cholesterol, HF; heart failure, HIV; human immunodeficiency virus, HOPE-3; heart outcomes prevention evaluation-3 trial, hs-CRP (HS-CRP); high-sensitivity C-reactive protein, LDL-C; low-density lipoprotein cholesterol, LLT; lipid-lowering therapy, Lp(a); lipoprotein(a), LVH; left ventricular hypertrophy, MI; myocardial infarction, NA; not available, NLI; national lead investigator, non-HDL-C; non–high-density lipoprotein cholesterol, PAD; peripheral arterial disease, RA; rheumatoid arthritis, REPRIEVE; randomized trial to prevent vascular events in HIV, SBP; systolic blood pressure, SEAS; simvastatin and ezetimibe in aortic stenosis, SHARP; study of heart and renal protection, TC; total cholesterol, TIA; transient ischaemic attack, TSH; thyroid-stimulating hormone, UACR; urine albumin-to-creatinine ratio, ULN; upper limit of normal, VESALIUS-CV; the effect of evolocumab in patients at high cardiovascular risk without prior myocardial infarction or stroke.

**Supplemental Table 6: Baseline characteristics of completed trials by annual placebo event rate**

| **Pooled trial-level characteristics*** | **Annual Placebo Event Rate** | | | **P value^#^** |
| --- | --- | --- | --- | --- |
|  | **<1%** | **≥1-<2%** | **≥2%** |  |
| No. of trials | 5 | 7 | 5 |  |
| Total N | 52,713 | 29,322 | 23,844 |  |
| Age (years) | 61.3 ± 9.3 | 65.6 ± 9.7 | 63.4 ± 11.1 | 0.549 |
| Female, n (%) | 19,008 (36.1) | 12,882 (43.9) | 7,502 (31.5) | <0.001 |
| LDL-C (mmol/L) | 3.24 | 3.43 | 3.71 | 0.501 |
| BMI (kg/m²) | 26.9 ± 4.8 | 28.5 ± 5.1 | 27.0 ± 4.9 | 0.321 |
| SBP (mmHg) | 135.6 ± 15.9 | 149.6 ± 21.4 | 141.9 ± 21.1 | 0.094 |
| DBP (mmHg) | 80.3 ± 9.5 | 87.4 ± 13.3 | 82.0 ± 11.8 | 0.186 |
| Hypertension, n (%) | 12,313 (35.3) | 23,854 (81.4) | 6,490 (44.5) | <0.001 |
| DM, n (%) | 2,555 (7.3) | 14,573 (55.4) | 4,030 (18.3) | <0.001 |
| Smoking, n (%) | 10,710 (20.3) | 5,905 (23.5) | 6,221 (26.1) | <0.001 |
| CKD, n (%) | NR | 301 (7.2) | 9,270 (100.0) |  |
| GFR (mL/min/1.73m²) | 74.0 ± 14.3 | 76.1 ± 25.3 | 33.6 ± 20.2 | 0.033 |
| Prior ASCVD, n (%) | 0 (0.0) | 1,858 (6.3) | 1,924 (8.1) | <0.001 |

*Values are weighted mean ± SD or n (%). Median/IQR converted to mean/SD using Wan et al. method for pooling.

^#^P values from weighted ANOVA (continuous variables) or chi-square test (categorical variables).

ASCVD; atherosclerotic cardiovascular disease, BMI; body mass index, CKD; chronic kidney disease, DBP; diastolic blood pressure, DM; diabetes mellitus, GFR; glomerular filtration rate, NR; not reported, SBP; systolic blood pressure,

**Supplemental Table 7: Annual placebo event rates for 5-year baseline risk strata and corresponding HR (99% CI) reported in CTT-2012 and their modification in the present analysis**

| **CTT-2012 meta-analysis** | | | | **THIS STUDY** | | |
| --- | --- | --- | --- | --- | --- | --- |
| **CTT endpoints in the 2012 meta-analysis publication** | | | | **CTT endpoints and modifications applied** | | |
| 5-year baseline risk category | Annual placebo event rate (%) | HR (99% CI) | RRR (99% CI) per 1 mmol/L reduction (%) | Annual placebo event rate (%) | HR (99% CI) | RRR (99% CI) per 1 mmol/L LDL-C reduction (%) |
| <5% | 0.53 | 0.61 (0.45-0.81) | 39 (19-55) | <1 | 0.61 (0.45-0.81) | 39 (19-55%) |
| ≥5% to <10% | 1.53 | 0.66 (0.57-0.77) | 34 (23-43) | ≥1-<2 | 0.66 (0.57-0.77) | 34 (23-43%) |
| ≥10% to <20% | 2.98 | 0.82 (0.72-0.93) | 18 (7-28%) | ≥2-<6 | 0.82 (0.73-0.91)* | 18 (9-27%) |
| ≥20% to <30% | 5.28 | 0.81 (0.65-1.01) | 19 (-1-35%) |  |  |  |
| ≥30% | 8.16 | 0.83 (0.58-1.18) | 17 (-18-42) | ≥6 | 0.83 (0.58-1.18) | 17 (-18-42%) |

^a^CTT endpoint: MVE defined as major coronary events (ie, non-fatal myocardial infarction or coronary death), strokes, or coronary revascularizations.

*HR (99%) CI obtained by inverse variance weighting of the hazard ratios corresponding to placebo event rate 2.98% and 5.28%

3P-MACE; major adverse cardiovascular event, CTT; cholesterol treatment trialists collaboration, HR; hazard ratio, MVE; major vascular event, RRR; relative risk reduction

**Supplemental Table 8: Annual placebo event rates and their corresponding standardized HR for CTT-2012 meta-analysis and the present analysis**

| **CTT-2012 meta-analysis (8 trials of statins)** | | **THIS STUDY (17 trials of statins and non-statins)** | |
| --- | --- | --- | --- |
| **CTT endpoint (MVE** : major coronary events (ie, non-fatal myocardial infarction or coronary death), strokes, or coronary revascularizations. | | **Endpoint from meta-regression (3P MACE:** non-fatal MI, non-fatal stroke, and cardiovascular death**)** | |
| Annual placebo event rate (%) | HR (99% CI) | Annual placebo event rate (%) | HR (95% CI) |
| 0.53 | 0.61 (0.45-0.81) | 1 | 0.64 (0.58-0.70) |
| 1.53 | 0.66 (0.57-0.77) | 2 | 0.75 (0.72, 0.79) |
| 2.98 | 0.82 (0.72-0.93) | 3 | 0.87 (0.81-0.93) |

3P-MACE; major adverse cardiovascular event, CTT; cholesterol treatment trialists collaboration, HR; hazard ratio, MVE; major vascular event, RRR; relative risk reduction

**Table 9A: Design, features, and population characteristics of the ongoing primary prevention trial included in this study.**

| **Trial** | **Total N** | **Trial site** | **Population** | **Age (years)** | **Female n (%)** | **Baseline LDL-C (mmol/L)** | **BMI (Kg/m^2^)** | **SBP (mmHg)** | **Hypertension, n (%)** | **Smoker, n (%)** |
| --- | --- | --- | --- | --- | --- | --- | --- | --- | --- | --- |
| STAREE  ^4^ | 9971 | Australia | Healthy individuals ≥70 years with TC ≤ 7.5mmol/L without prior CVD (defined as myocardial infarction, angina, coronary artery angioplasty and/or stenting, coronary artery bypass grafting surgery, heart failure, stroke, transient ischemic attack, carotid stenosis, abdominal aortic aneurysm, and peripheral vascular disease), diabetes, dementia, or other life‐limiting conditions, and living independently | 74.7 (4.5) | 5174  (52) | 3.27±0.72 | 27.0  (24.3-30.1) | 136±16 | 4311  (43) | 4501  (45) |

CVD; cardiovascular disease, STAREE; statins in reducing events in the elderly.

**Table 9B: Key predicted outcomes of the ongoing primary prevention trial included in the current analysis**

| **Trial** | **Median follow-up (years)** | **Percentage LDL-C reduction** | **Baseline LDL-C (mmol/L)** | **Average LDL-C reduction (mmol/L)** | **Annual placebo event rate in CTT-2012, considered for predicting RRR** | **Predicted RRR (95% CI) for 3P MACE** |
| --- | --- | --- | --- | --- | --- | --- |
| STAREE ^4^ | 6 | 40% | 3.27 | 1.31 | <1% | 48% |
|  |  |  |  |  | ≥1%-<2% | 42% |
|  |  |  |  |  | ≥2%-<6% | 23% |
|  |  |  |  |  | ≥6% | 22% |

*Percentage LDL-C reduction was based on a large statin meta-analysis (VOYAGER) by Björn W Karlson et al. ^5^

3P-MACE; 3 point major adverse cardiovascular event, CTT-2012; Cholesterol Treatment Trialists’ Collaboration, 2012, STAREE; statins in reducing events in the elderly, VOYAGER; individual patient meta-analysis of statin therapy in at-risk groups: effects of rosuvastatin, atorvastatin and simvastatin

Studies from databases/registers **(n = 2183)**

EMBASE (n = 1162)

MEDLINE (n = 611)

CENTRAL (n = 410)

References from other sources **(n = 0)**

Citation searching (n = 0)

Grey literature (n = 0)

**Identification**

References removed **(n = 472)**

Duplicates identified manually (n = 19)

Duplicates identified by Covidence (n = 453)

Marked as ineligible by automation tools (n = 0)

Other reasons (n = 0)

Studies screened **(n = 1711)**

Studies sought for retrieval **(n = 68)**

Studies excluded **(n = 1643)**

Studies excluded **(n = 50)**

1. Post-hoc primary prevention analyses from trials included in CTT-2012 or this review (n = 16)

2. Post-hoc primary prevention analyses, non-MACE endpoints (n = 9)

3. Mixed primary and secondary prevention; primary prevention not reported separately or secondary prevention ≥ 20% (n = 19)

4. MACE end point not well reported (n = 2)

5. Ongoing trials without published baseline characteristics (n = 2)

6. Trials with <1000 enrolled participants (n=1)

7. Trial with renal transplant patients (n=1)

Studies not retrieved **(n = 0)**

**Screening**

Studies assessed for eligibility **(n = 68)**

**Included**

Studies included in review **(n = 18)**

**Supplemental Figure 1:** PRISMA flow diagram showing selection of eligible trials.

CENTRAL; Cochrane Central Register of Controlled Trials, CTT-2012;Cholesterol Treatment Trialists’ Collaboration, 2012, MACE; major adverse cardiovascular event.

**Supplemental Figure 2**: Standardized RRR of trials not included in CTT-2012 across baseline risk thresholds (event rates) based on CTT-2012 estimates. Regression lines represent the RRR per 1 mmol/L LDL-C lowering across annualized placebo event rates, corresponding to 5-year baseline cardiovascular risk categories among primary prevention participants in the CTT-2012. Squares represent individual trials, with size proportional to study weight. Colours indicate placebo event rate categories.

*CLEAR Outcomes and VESALIUS-CV estimates correspond to the standardized CTT-2012 endpoint reported by Lincoff et al ^6^and Marston^7^, respectively. Estimates for all other trials are for standardized 3P-MACE.

ALLHAT-LLT; antihypertensive and lipid-lowering treatment to prevent heart attack trial–lipid-lowering trial, CLEAR Outcomes; cholesterol lowering via bempedoic acid, an ACL-inhibiting regimen, EWTOPIA 75; ezetimibe lipid-lowering trial on prevention of atherosclerotic cardiovascular disease in 75 or older, HOPE-3; heart outcomes prevention evaluation-3 trial, REPRIEVE; randomized trial to prevent vascular events in HIV, SEAS; simvastatin and ezetimibe in aortic stenosis, SHARP; study of heart and renal protection, VESALIUS-CV; The Effect of Evolocumab in Patients at High Cardiovascular Risk without Prior Myocardial Infarction or Stroke.

**Supplemental Figure 3:** RRR of trials not included in CTT-2012 with 0% ASCVD prevalence across baseline risk thresholds (event rates) based on CTT-2012 estimates. Regression lines represent the RRR per 1 mmol/L LDL-C lowering across annualized placebo event rates, corresponding to 5-year baseline cardiovascular risk categories among primary prevention participants in the CTT-2012. Squares represent individual trials, with size proportional to study weight. Colors indicate placebo event rate categories.

*CLEAR Outcomes and VESALIUS-CV estimates correspond to the unstandardized CTT-2012 endpoint reported by Lincoff et al ^6^and Marston^7^, respectively. Estimates for all other trials are for unstandardized 3P-MACE.

ALLHAT-LLT; antihypertensive and lipid-lowering treatment to prevent heart attack trial–lipid-lowering trial, CLEAR Outcomes; cholesterol lowering via bempedoic acid, an ACL-inhibiting regimen, HOPE-3; heart outcomes prevention evaluation-3 trial, REPRIEVE; randomized trial to prevent vascular events in HIV, SEAS; simvastatin and ezetimibe in aortic stenosis, TRACE-RA; Trial of Atorvastatin for the Primary Prevention of Cardiovascular Events in Patients with Rheumatoid Arthritis, VESALIUS-CV; The Effect of Evolocumab in Patients at High Cardiovascular Risk without Prior Myocardial Infarction or Stroke.

**Figure S4B**

**Figure S4A**


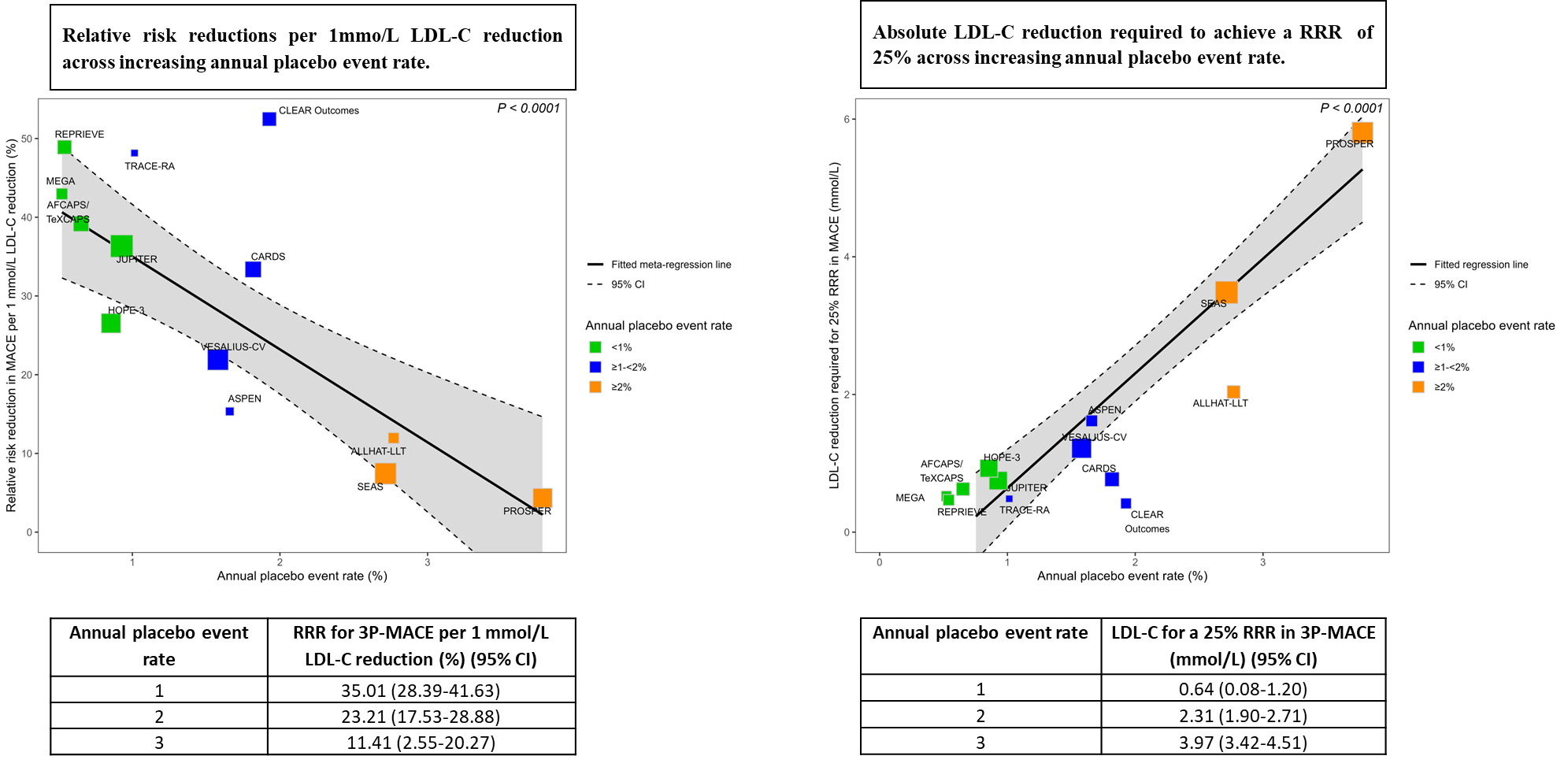


**Supplemental Figure 4:** Meta-regression of RRR (y axis) in 3PMACE standardized per 1 mmol/L LDL-C lowering as a function of annualized placebo event rate (x axis) (S4A); absolute LDL-C required to achieve a 25% RRR (S4B) across increasing annualized placebo event rate in trials without prevalent established ASCVD.

Squares represent individual trials, with size proportional to study weight. Colors indicate placebo event rate categories. The solid line shows the fitted regression line, and dashed lines indicate the 95% confidence intervals.

AFCAPS/TeXCAPS; Air Force/Texas Coronary Atherosclerosis Prevention Study, ALLHAT-LLT; Antihypertensive and Lipid-Lowering Treatment to Prevent Heart Attack Trial-Lipid Lowering Trial, ASCOT-LLA; Anglo-Scandinavian Cardiac Outcomes Trial-Lipid Lowering Arm, ASPEN; Atorvastatin Study for Prevention of Coronary Heart Disease Endpoints in Non-Insulin-Dependent Diabetes Mellitus, CARDS; Collaborative Atorvastatin Diabetes Study, CLEAR Outcomes; Cholesterol Lowering via Bempedoic Acid, an ACL-Inhibiting Regimen Outcomes, EWTOPIA 75; Ezetimibe Lipid-Lowering Trial On Prevention of Atherosclerosis in 75 or Older; HOPE-3; Heart Outcomes Prevention Evaluation-3, JUPITER; Justification for the Use of Statins in Prevention: an Intervention Trial Evaluating Rosuvastatin, MEGA; Management of Elevated Cholesterol in the Primary Prevention Group of Adult Japanese, PROSPER; PROspective Study of Pravastatin in the Elderly at Risk, REPRIEVE; Randomized Trial to Prevent Vascular Events in HIV, SEAS; Simvastatin and Ezetimibe in Aortic Stenosis, SHARP; Study of Heart and Renal Protection, TRACE-RA; Trial of Atorvastatin for the Primary Prevention of Cardiovascular Events in Patients with Rheumatoid Arthritis, VESALIUS-CV; The Effect of Evolocumab in Patients at High Cardiovascular Risk without Prior Myocardial Infarction or Stroke, VESALIUS-CV; The Effect of Evolocumab in Patients at High Cardiovascular Risk without Prior Myocardial Infarction or Stroke, WOSCOPS; West of Scotland Coronary Prevention Study.


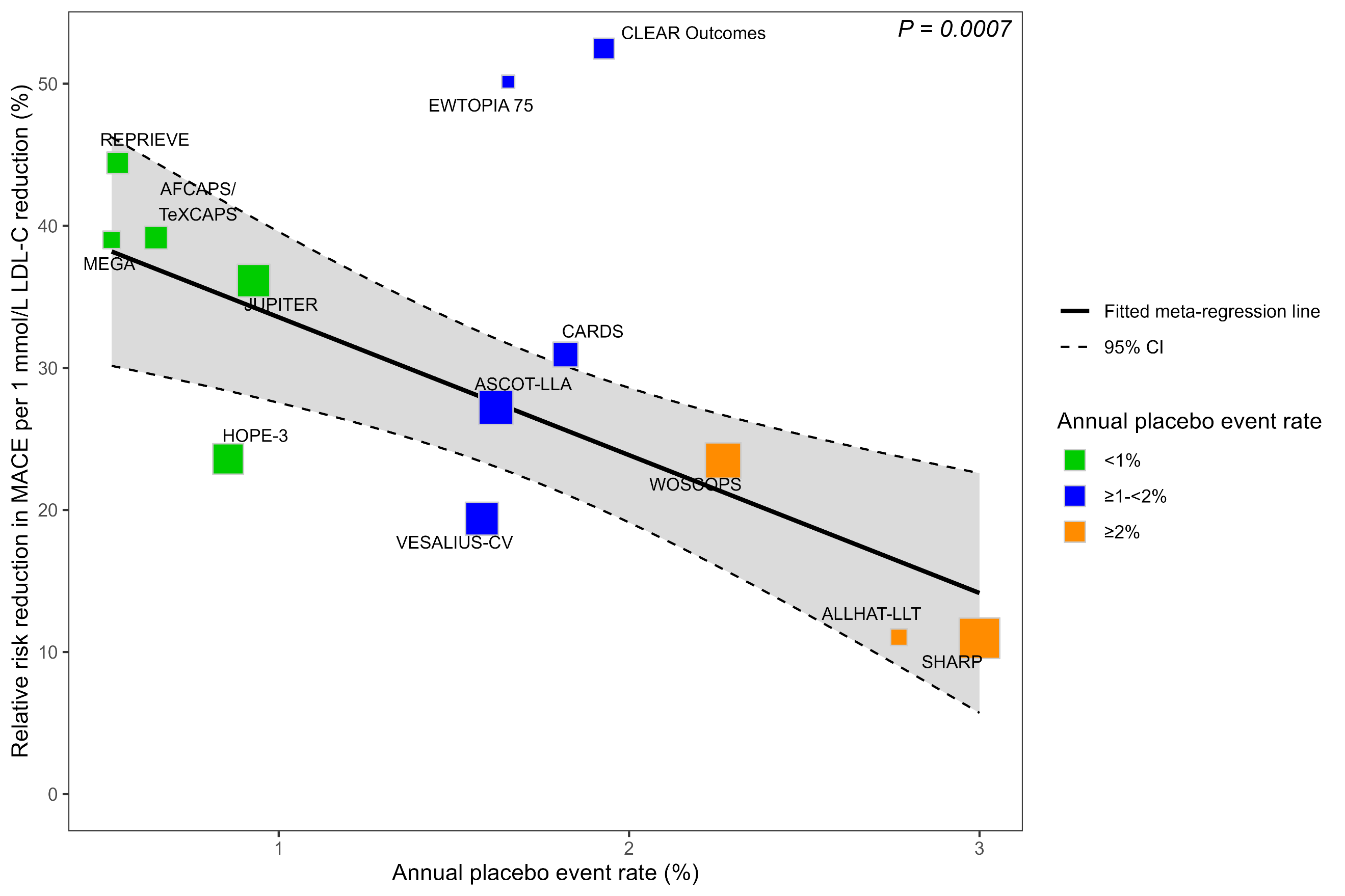


**Supplemental Figure 5**: Meta-regression of RRR (y axis) in 3PMACE standardized per 1 mmol/L LDL-C lowering as a function of annualized placebo event rate (x axis), among trials reporting between-group change in LDL-C only at 1 year.

AFCAPS/TeXCAPS; Air Force/Texas Coronary Atherosclerosis Prevention Study, ALLHAT-LLT; Antihypertensive and Lipid-Lowering Treatment to Prevent Heart Attack Trial-Lipid Lowering Trial, ASCOT-LLA; Anglo-Scandinavian Cardiac Outcomes Trial-Lipid Lowering Arm, CARDS; Collaborative Atorvastatin Diabetes Study, CLEAR Outcomes; Cholesterol Lowering via Bempedoic Acid, an ACL-Inhibiting Regimen Outcomes, EWTOPIA 75; Ezetimibe Lipid-Lowering Trial On Prevention of Atherosclerosis in 75 or Older; HOPE-3; Heart Outcomes Prevention Evaluation-3, JUPITER; Justification for the Use of Statins in Prevention: an Intervention Trial Evaluating Rosuvastatin, MEGA; Management of Elevated Cholesterol in the Primary Prevention Group of Adult Japanese, REPRIEVE; Randomized Trial to Prevent Vascular Events in HIV, SHARP; Study of Heart and Renal Protection, TRACE-RA; Trial of Atorvastatin for the Primary Prevention of Cardiovascular Events in Patients with Rheumatoid Arthritis, VESALIUS-CV; The Effect of Evolocumab in Patients at High Cardiovascular Risk without Prior Myocardial Infarction or Stroke, VESALIUS-CV; The Effect of Evolocumab in Patients at High Cardiovascular Risk without Prior Myocardial Infarction or Stroke, WOSCOPS; West of Scotland Coronary Prevention Study.


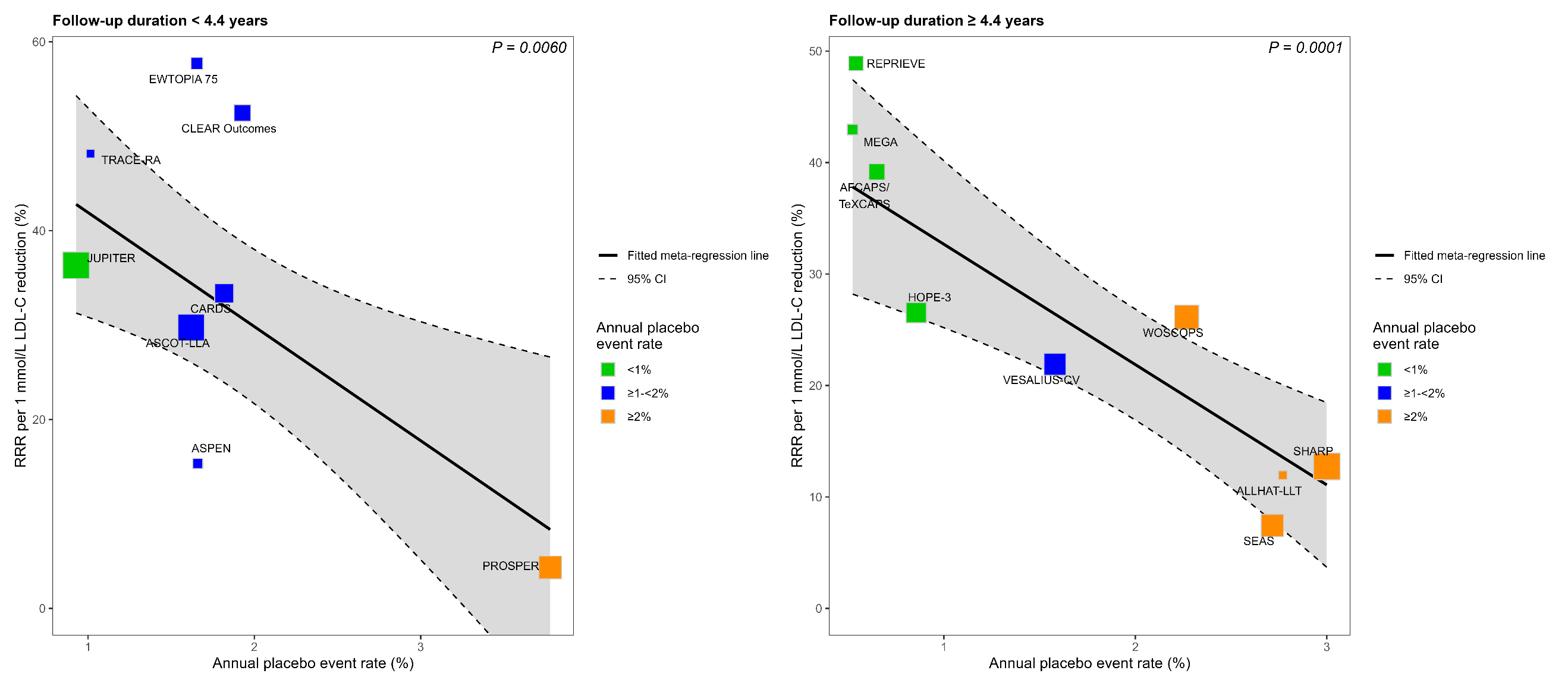


**Figure S6A**

**Figure S6B**


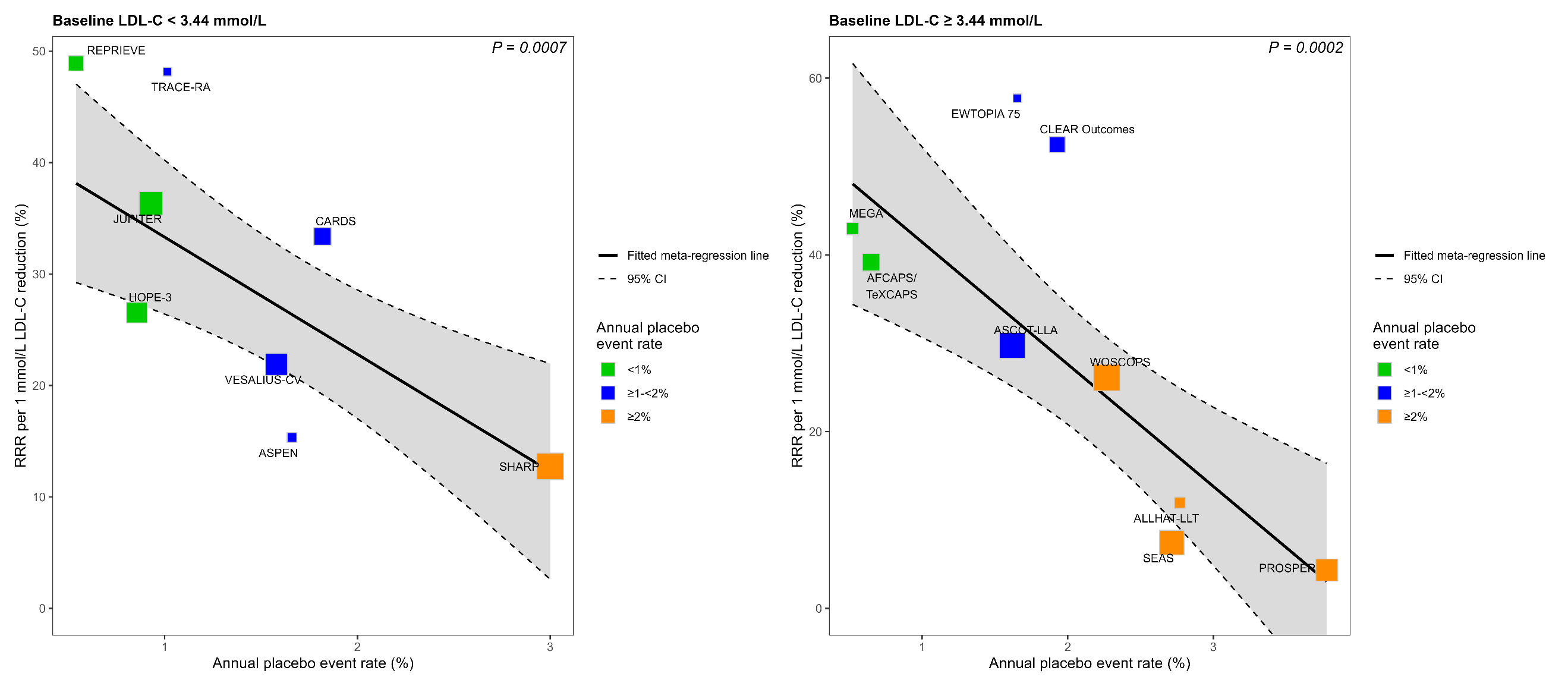


**Supplemental Figure 6**: Meta-regression of RRR (y axis) in 3PMACE standardized per 1 mmol/L LDL-C lowering as a function of annualized placebo event rate (x axis), stratified by median follow-up duration **(S6A)** and baseline LDL-C **(S6B)**.

AFCAPS/TeXCAPS; Air Force/Texas Coronary Atherosclerosis Prevention Study, ALLHAT-LLT; Antihypertensive and Lipid-Lowering Treatment to Prevent Heart Attack Trial-Lipid Lowering Trial, ASCOT-LLA; Anglo-Scandinavian Cardiac Outcomes Trial-Lipid Lowering Arm, CARDS; Collaborative Atorvastatin Diabetes Study, CLEAR Outcomes; Cholesterol Lowering via Bempedoic Acid, an ACL-Inhibiting Regimen Outcomes, EWTOPIA 75; Ezetimibe Lipid-Lowering Trial On Prevention of Atherosclerosis in 75 or Older; HOPE-3; Heart Outcomes Prevention Evaluation-3, JUPITER; Justification for the Use of Statins in Prevention: an Intervention Trial Evaluating Rosuvastatin, MEGA; Management of Elevated Cholesterol in the Primary Prevention Group of Adult Japanese, REPRIEVE; Randomized Trial to Prevent Vascular Events in HIV, SHARP; Study of Heart and Renal Protection, TRACE-RA; Trial of Atorvastatin for the Primary Prevention of Cardiovascular Events in Patients with Rheumatoid Arthritis, VESALIUS-CV; The Effect of Evolocumab in Patients at High Cardiovascular Risk without Prior Myocardial Infarction or Stroke, VESALIUS-CV; The Effect of Evolocumab in Patients at High Cardiovascular Risk without Prior Myocardial Infarction or Stroke, WOSCOPS; West of Scotland Coronary Prevention Study.


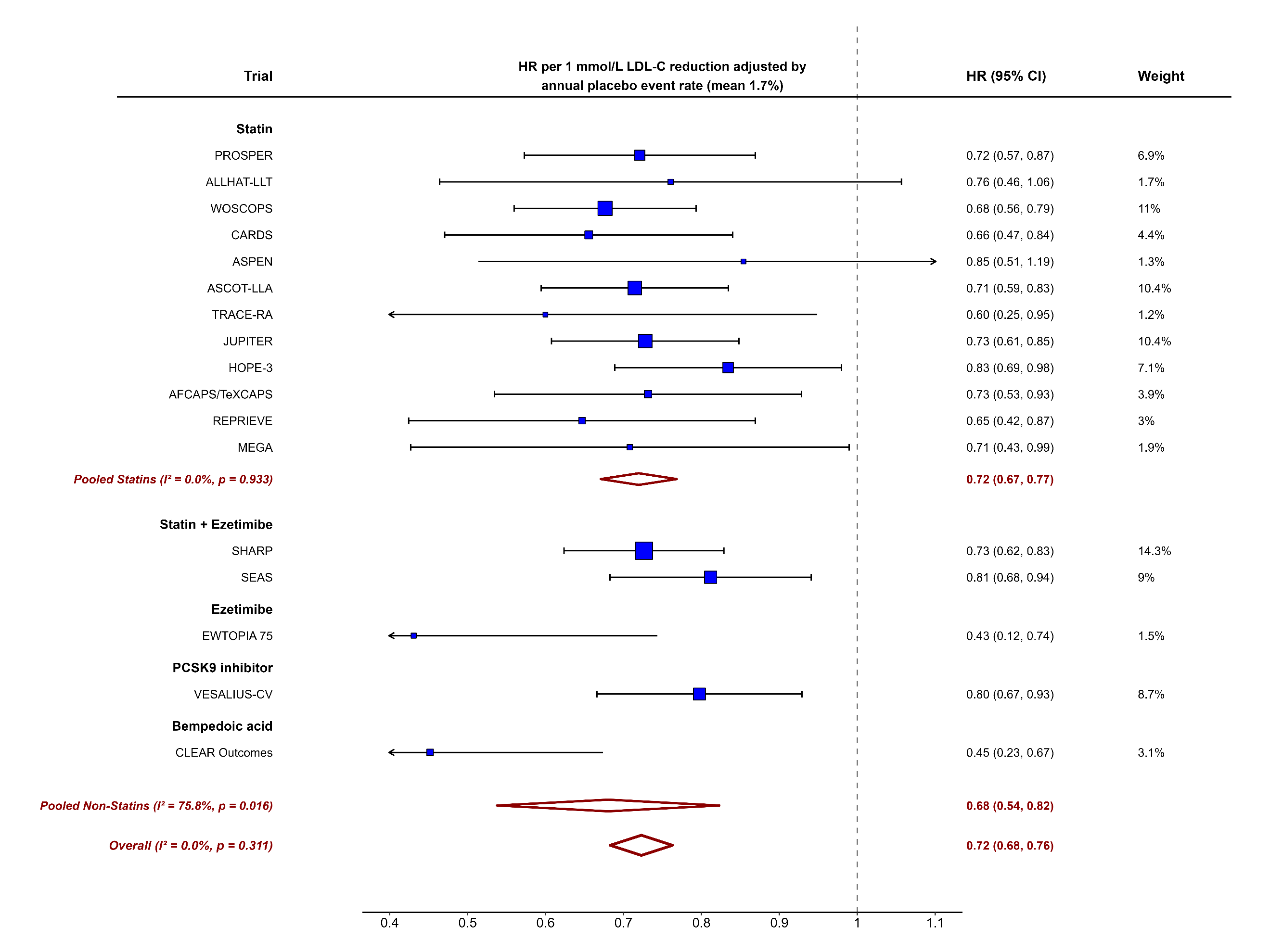


**Supplemental Figure 7**: Meta-analysis of HR for 3P-MACE standardized per mmol/L and adjusted to the mean annual placebo event rate (1.7%) by LLT class. Each square represents individual RCT with size proportionate to the inverse variance weight. CIs extending beyond the displayed range are indicated by arrows. The diamonds represent the pooled estimates for statins, non-statin trials and for the overall combination of all trials.

3P-MACE; 3 point major adverse cardiovascular event, AFCAPS/TeXCAPS; Air Force/Texas Coronary Atherosclerosis Prevention Study, ALLHAT-LLT; Antihypertensive and Lipid-Lowering Treatment to Prevent Heart Attack Trial-Lipid Lowering Trial, ASCOT-LLA; Anglo-Scandinavian Cardiac Outcomes Trial-Lipid Lowering Arm, ASPEN; Atorvastatin Study for Prevention of Coronary Heart Disease Endpoints in Non-Insulin-Dependent Diabetes Mellitus, CARDS; Collaborative Atorvastatin Diabetes Study, CI; confidence interval, CLEAR Outcomes; Cholesterol Lowering via Bempedoic Acid, an ACL-Inhibiting Regimen Outcomes, EWTOPIA 75; Ezetimibe Lipid-Lowering Trial On Prevention of Atherosclerosis in 75 or Older; HR; hazard ratio, HOPE-3; Heart Outcomes Prevention Evaluation-3, JUPITER; Justification for the Use of Statins in Prevention: an Intervention Trial Evaluating Rosuvastatin, LLT; lipid lowering therapy, MEGA; Management of Elevated Cholesterol in the Primary Prevention Group of Adult Japanese, PROSPER; PROspective Study of Pravastatin in the Elderly at Risk, REPRIEVE; Randomized Trial to Prevent Vascular Events in HIV, SEAS; Simvastatin and Ezetimibe in Aortic Stenosis, SHARP; Study of Heart and Renal Protection, TRACE-RA; Trial of Atorvastatin for the Primary Prevention of Cardiovascular Events in Patients with Rheumatoid Arthritis, VESALIUS-CV; The Effect of Evolocumab in Patients at High Cardiovascular Risk without Prior Myocardial Infarction or Stroke, WOSCOPS; West of Scotland Coronary Prevention Study.


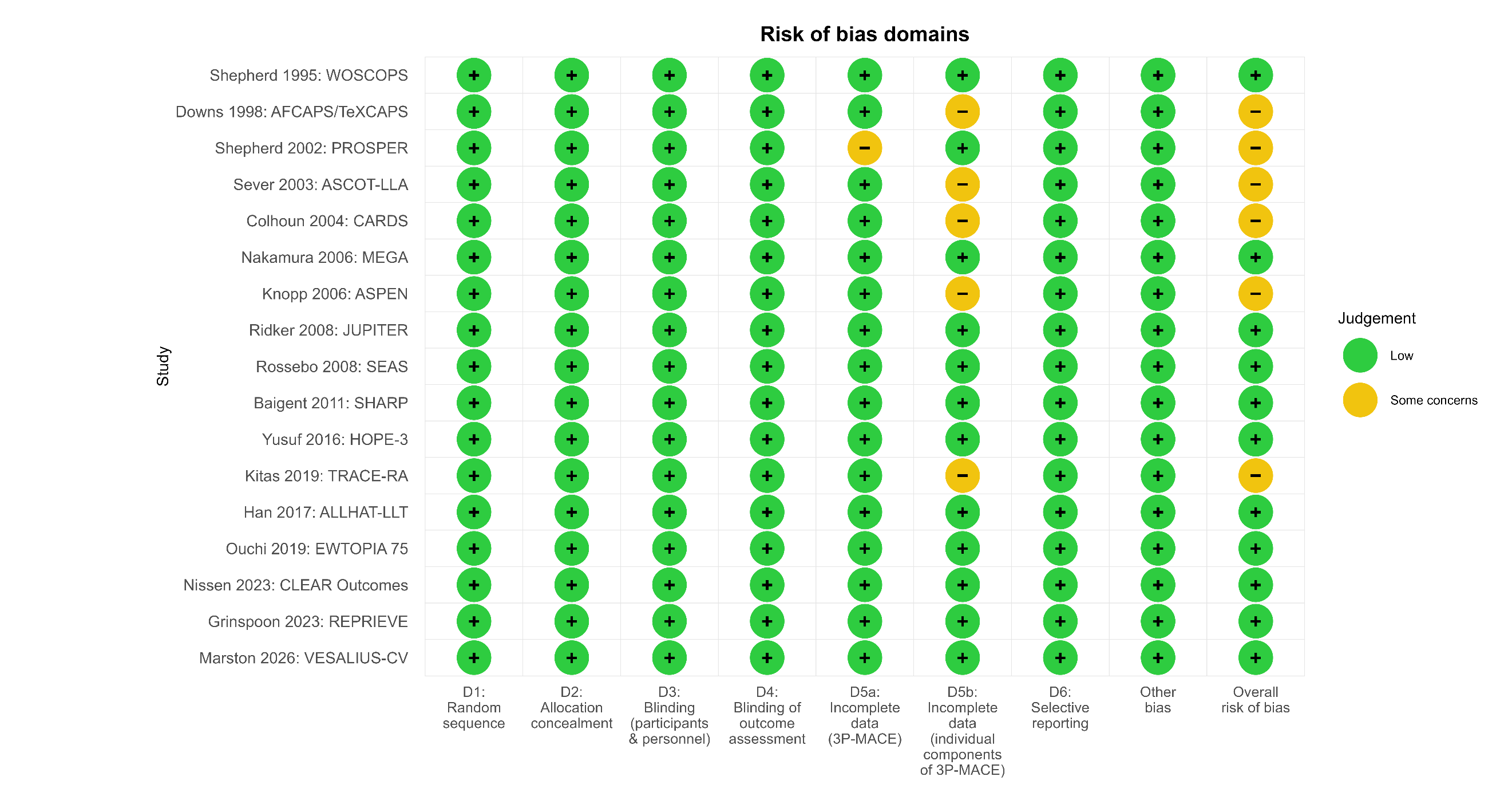


**Supplemental Figure 8:** Risk-of-bias assessment across completed trials (Cochrane RoB-1). Traffic-light plot showing domain-level judgements for each study: D1, random sequence generation; D2, allocation concealment; D3, blinding of participants and personnel; D4, blinding of outcome assessment; D5a, incomplete outcome data (3P-MACE); D5b, incomplete outcome data (3P-MACE components); D5c, incomplete outcome data (all outcomes); D6, selective reporting; and other bias.

3P-MACE; 3-point Major adverse cardiovascular event, AFCAPS/TeXCAPS; Air Force/Texas Coronary Atherosclerosis Prevention Study, ALLHAT-LLT; Antihypertensive and Lipid-Lowering Treatment to Prevent Heart Attack Trial-Lipid Lowering Trial, ASCOT-LLA; Anglo-Scandinavian Cardiac Outcomes Trial-Lipid Lowering Arm, ASPEN; Atorvastatin Study for Prevention of Coronary Heart Disease Endpoints in Non-Insulin-Dependent Diabetes Mellitus, CARDS; Collaborative Atorvastatin Diabetes Study, CLEAR Outcomes; Cholesterol Lowering via Bempedoic Acid, an ACL-Inhibiting Regimen Outcomes, EWTOPIA 75; Ezetimibe Lipid-Lowering Trial On Prevention of Atherosclerosis in 75 or Older; HOPE-3; Heart Outcomes Prevention Evaluation-3, JUPITER; Justification for the Use of Statins in Prevention: an Intervention Trial Evaluating Rosuvastatin, MEGA; Management of Elevated Cholesterol in the Primary Prevention Group of Adult Japanese, PROSPER; PROspective Study of Pravastatin in the Elderly at Risk, REPRIEVE; Randomized Trial to Prevent Vascular Events in HIV, SEAS; Simvastatin and Ezetimibe in Aortic Stenosis, SHARP; Study of Heart and Renal Protection, TRACE-RA; Trial of Atorvastatin for the Primary Prevention of Cardiovascular Events in Patients with Rheumatoid Arthritis, VESALIUS-CV; The Effect of Evolocumab in Patients at High Cardiovascular Risk without Prior Myocardial Infarction or Stroke, WOSCOPS; West of Scotland Coronary Prevention Study.

**Supplemental Figure 9:** Predicted RRR for CTT endpoint RRR for ongoing trial **(S9A)** and observed RRR for completed trials **(S9B)** by risk threshold (event rates) from CTT-2012. Regression lines represent the RRR per 1 mmol/L LDL-C lowering across annualized placebo event rates, corresponding to 5-year baseline cardiovascular risk categories among primary prevention participants in the CTT-2012. Squares represent individual trials, with size proportional to study weight. Colours indicate placebo event rate categories.

*CLEAR Outcomes and VESALIUS-CV estimates correspond to the unstandardized CTT-2012 endpoint reported by Lincoff et al ^6^and Marston^7^, respectively. Estimates for all other trials are for unstandardized 3P-MACE.

ALLHAT-LLT; antihypertensive and lipid-lowering treatment to prevent heart attack trial–lipid-lowering trial, CLEAR Outcomes; cholesterol lowering via bempedoic acid, an ACL-inhibiting regimen, EWTOPIA 75; ezetimibe lipid-lowering trial on prevention of atherosclerotic cardiovascular disease in 75 or older, HOPE-3; heart outcomes prevention evaluation-3 trial, REPRIEVE; randomized trial to prevent vascular events in HIV, SEAS; simvastatin and ezetimibe in aortic stenosis, SHARP; study of heart and renal protection, STAREE; statins in reducing events in the elderly. REPRIEVE; Randomized Trial to Prevent Vascular Events in HIV, SEAS; simvastatin and ezetimibe in aortic stenosis, SHARP; study of heart and renal protection, VESALIUS-CV; The Effect of Evolocumab in Patients at High Cardiovascular Risk without Prior Myocardial Infarction or Stroke.

**Supplemental Figure 10:** Predicted RRR for CTT endpoint for ongoing trial and observed RRR for completed trials by each risk category (event rate) from CTT-2012

Regression lines represent the RRR per 1 mmol/L LDL-C lowering across annualized placebo event rates, corresponding to 5-year baseline cardiovascular risk categories among primary prevention participants in the CTT-2012. Squares represent individual trials, with size proportional to study weight. Colors indicate placebo event rate categories.

*CLEAR Outcomes and VESALIUS-CV estimates correspond to the unstandardized CTT-2012 endpoint reported by Lincoff et al ^6^and Marston^7^, respectively. Estimates for all other trials are for unstandardized 3P-MACE.

ALLHAT-LLT; antihypertensive and lipid-lowering treatment to prevent heart attack trial–lipid-lowering trial, CLEAR Outcomes; cholesterol lowering via bempedoic acid, an ACL-inhibiting regimen, EWTOPIA 75; ezetimibe lipid-lowering trial on prevention of atherosclerotic cardiovascular disease in 75 or older, HOPE-3; heart outcomes prevention evaluation-3 trial, REPRIEVE; randomized trial to prevent vascular events in HIV, SEAS; simvastatin and ezetimibe in aortic stenosis, SHARP; study of heart and renal protection, STAREE; statins in reducing events in the elderly, TRACE-RA; Trial of Atorvastatin for the Primary Prevention of Cardiovascular Events in Patients with Rheumatoid Arthritis, VESALIUS-CV; The Effect of Evolocumab in Patients at High Cardiovascular Risk without Prior Myocardial Infarction or Stroke.

**References**

1. Wan X, Wang W, Liu J, et al. Estimating the sample mean and standard deviation from the sample size, median, range and/or interquartile range. BMC Med Res Methodol. 2014;14:135.

2. Cholesterol Treatment Trialists C. The effects of lowering LDL cholesterol with statin therapy in people at low risk of vascular disease: meta-analysis of individual data from 27 randomised trials. The Lancet. 2012;380(9841):581-590.

3. Ray KK, Gunn LH, Conde LG, et al. Estimating potential cardiovascular health benefits of improved population level control of LDL cholesterol through a twice-yearly siRNA-based approach: A simulation study of a health-system level intervention. Atherosclerosis. 2024;391:117472.

4. Zoungas S, Moran C, Curtis AJ, et al. Baseline Characteristics of Participants in STAREE: A Randomized Trial for Primary Prevention of Cardiovascular Disease Events and Prolongation of Disability‐Free Survival in Older People. Journal of the American Heart Association. 2024;13(22):e036357.

5. Karlson BW, Palmer MK, Nicholls SJ, et al. Attainment of the anticipated &gt;=50% reduction in LDL-C in the four ACC/AHA guidelines statin benefit groups: A voyager meta-analysis. Atherosclerosis. 2014;235(2):e11-e12.

6. Lincoff AM, Ray KK, Sasiela WJ, et al. Comparative Cardiovascular Benefits of Bempedoic Acid and Statin Drugs. JACC. 2024;84(2):152-162.

7. Marston NA, Bohula EA, Bhatia AK, et al. Evolocumab to Reduce First Major Cardiovascular Events in Patients Without Known Significant Atherosclerosis and With Diabetes: Results From the VESALIUS-CV Trial. JAMA. 2026.
